# Integration of Biochemical and Electrical Signaling-Multiscale Model of the Medium Spiny Neuron of the Striatum

**DOI:** 10.1371/journal.pone.0066811

**Published:** 2013-07-03

**Authors:** Michele Mattioni, Nicolas Le Novère

**Affiliations:** 1 European Molecular Biology Laboratory-The European Bioinformatics Institute (EMBL-EBI), Hinxton, Cambridge, United Kingdom; 2 Babraham Institute, Babraham, Cambridge, United Kingdom; Neuroscience Campus Amsterdam, VU University, The Netherlands

## Abstract

Neuron behavior results from the interplay between networks of biochemical processes and electrical signaling. Synaptic plasticity is one of the neuronal properties emerging from such an interaction. One of the current approaches to study plasticity is to model either its electrical aspects or its biochemical components. Among the chief reasons are the different time scales involved, electrical events happening in milliseconds while biochemical cascades respond in minutes or hours. In order to create multiscale models taking in consideration both aspects simultaneously, one needs to synchronize the two models, and exchange relevant variable values. We present a new event-driven algorithm to synchronize different neuronal models, which decreases computational time and avoids superfluous synchronizations. The algorithm is implemented in the TimeScales framework. We demonstrate its use by simulating a new multiscale model of the Medium Spiny Neuron of the Neostriatum. The model comprises over a thousand dendritic spines, where the electrical model interacts with the respective instances of a biochemical model. Our results show that a multiscale model is able to exhibit changes of synaptic plasticity as a result of the interaction between electrical and biochemical signaling. Our synchronization strategy is general enough to be used in simulations of other models with similar synchronization issues, such as networks of neurons. Moreover, the integration between the electrical and the biochemical models opens up the possibility to investigate multiscale process, like synaptic plasticity, in a more global manner, while taking into account a more realistic description of the underlying mechanisms.

## Introduction

A model can be considered multiscale when at least one of these conditions is true: (i) the variables used in the model span different orders of magnitude either in time or space, e.g. from milliseconds to minutes, or from nanometers to millimeters; (ii) parts of the model are simulated with different time and/or spatial resolutions, influencing each other through a systematic exchange of variables [1]. In computational neuroscience, multiscale modeling is necessary on various fronts. While spatial stochastic simulations of a femtoliter and a few thousands molecules are computationally tractable and can provide good approximations of the dynamics happening within a spine of a neuron [2], they are hardly usable for an entire neuron. Neither are they desirable if we only need to understand the electrical properties of the cell. A possible solution could be to integrate spatial simulation of small compartments with multicompartment Hodgkin-Huxley based models. These multiscale models could help understand the overall electrical behavior of the neuron, keeping the ability of zooming in a specific part of the neuron, using the spatial simulations to gain detailed understanding of the biochemical reactions occurring there. Modeling large neuronal networks poses the same problem. Simulating highly detailed multicompartment neurons require substantial computational power, making them a sub-optimal choice to model significant parts of the brain [3]. Dedicated hardware and software are needed, such as the supercomputer IBM BlueGene/L, used by the Blue Brain project [4]. Alternatively, a possible solution is to model large networks using integrate and fire neurons [5], [6], and integrate them with multicompartment models only in the cases where a detailed understanding of electrophysiology at subcellular level is needed.

The timescale issue also occurs when modeling the interactions between electrical and biochemical processes. Both domains could be modeled together using Ordinary Differential Equations (ODEs) or Partial Differential Equations (PDEs). However, the difference of characteristic timescales between the fast and slow reactions (sub-second for electrical processes, multi-minutes for the biochemical ones) will impact the efficiency of the numerical solver. Moreover, the electrical and the biochemical system mostly interact when a certain type of event happens, like a stimulation, and can be considered independent otherwise. This makes continuous integration an unnecessary burden.

Synaptic plasticity is the biological process that modulates the strength of the connections between two neurons at the synaptic level, based on prior activity. Plasticity can be defined using a time relationship, which compares different responses of the synapse to the same *stimuli* (such as post-synaptic potentials) over time. Several factors influence the strength of the synapse, such as the number, state and type of receptors present in the postsynaptic density (PSD) [7]. Long Term Potentiation (LTP), reflecting an increase of synaptic strength, and Long Term Depression (LTD), reflecting a decrease in synaptic strength, are two widespread forms of synaptic plasticity. The timescales of the events involved in these biological processes span from microseconds to minutes, or even up to hours and days when translation and transcription occur [8]. Temporal distinction is used to classify Long Term Memory (LTM) into two groups: Early-Long Term Memory (E-LTM), where there is no protein translation, and the maximum timescale is usually in the range of minutes, and Late-Long Term Memory (L-LTM), when the synthesis of new proteins is required and the range is of hours or longer [9].

Only a multiscale modeling approach can address the wide range of timescales, involved in spatial, electrical and biochemical processes [10] The integration of different submodels in a single larger model is becoming more frequent in Systems Biology, thanks in part to standards that are providing guidelines to approach the problem [11] Simple models can indeed be created independently, and later joined together in a more complex one [12]. This approach is feasible with models sharing the underlying mathematical framework, for instance process representation in chemical kinetics models of biochemical pathways. However, the same strategy cannot be applied to merge models of biochemical and electrical signaling. These two aspects of neuronal function are usually modeled using two different mathematical approaches, which are solved using different methods.

According to the Clustered Plasticity Hypothesis (CPH), proposed by Govindarajan et al. [13], neighboring spines have the ability to influence each other, even if not all are directly stimulated. The computational unit is therefore not the spine alone as proposed by Matsuzaki et al. [14], but the dendritic branch where the stimulated spines reside. It has to be noted that Govindarajan et al. [13] are referring to an ensemble of stimulated spines. Therefore each spine itself still contributes to the synaptic plasticity, either being in a L-LTP or L-LTD status, but also integrates with the others. This creates specialized engrams, composed of precise set of spines. An engram is a persistent change in the brain that is formed in response to a stimulus, and is the neuronal substrate for a memory (also known as a memory trace). In a recent paper, Govindarajan et al. [9] have demonstrated this neighboring influence on a hippocampal slices. Among the proposed mechanisms by which neighboring spines could influence each other are: (i) the supralinear integration of inputs [15], [16], (ii) the diffusion of proteins like Ras [17]–[19], which are able to migrate from one stimulated spine to neighboring ones.

Given the continuous interaction between the biochemical and the electrical processes, a multiscale model of a neuron with explicit descriptions of spines would help to understand how both processes interact, which timescales are involved, what are the consequences on a single spine, and help assess the interactions of a stimulated spine with the neighboring ones. In this manuscript, we use this approach to study the Medium Spiny Neuron (MSN) of the striatum.

## Results

### TimeScales Framework, a General Approach to Integrate Biochemical and Electrical Modeling

TimeScales works as a meta-simulator, able to run a collection of two or more different models as an holistic system, connecting them together and taking care of the synchronization. The modeling frameworks used by the different components can be different, resulting in an *Hybrid Model*. To perform an optimal synchronization, two main types of information must be available to the synchronization algorithm: (i) the time of events, (ii) the variables to exchange. To achieve this objective, the simulators need to expose three methods: a *set* method, to export the value of a variable, a *get* method, to import the value of a variable and a *step* method, to advance the simulator forward in time.

The algorithm synchronizes two simulators only when an event affect both, otherwise it runs the simulators separately. The main idea behind the algorithm is to run the two simulators apart from each other as long as possible, and to minimize the time spent in the time consuming synchronization loop. Since the synchronization is event-driven, the instant an event happens is the key information for the system. Events can be known *a priori*, as it is the case in this paper where stimulations are defined, or events can emerge from the simulation of the system, as would be the case for example in a neuronal network. Currently *TimeScales* focuses only on the first case, but we suggest how it is possible to extend the framework to operate in a situation where the events are generated during the simulation. In the first case, the events can be presorted chronologically in a lookup table which is checked to decide if a synchronization has to be performed or not. The first chronological event will be the point where the synchronization will happen. The event will be removed from the queue, and the procedure will be repeated until the end of the time of the simulation, as shown in pseudo code below.

   events_queue = create_events_queue()

   while (events_queue ! = []):

     event_time = events_queue.pop()

     run_separately(event_time)

     synchronize(event_time)

   run_separately(tstop)

### Deciding the Model Hierarchy

In a master-slave configuration, the master process distributes the work to the slave processes, which execute it and return the result back to the master. The master then assembles the complete result. This strategy is usually adopted to parallelize the execution of complex programs, see for instance the MPI packages [20], [21]. The idea of a master-slave configuration for multiscale modeling has been used for example by Cao et al. [22], where the analysis of a tunnel structure has been modeled using two different grids, composed by master and slaves nodes at different resolutions, where the force applied to the master node is the result of the forces applied on the slaves nodes.

It is possible to think of the TimeScales framework as a master-slave configuration where the event-driven algorithm acts as the master process, coordinating and integrating the result coming from the slaves, which are the simulators to synchronize. The main difference with a classic master-slave configuration is the type of computation performed by the simulators. In the TimeScales framework, a precise hierarchy must be respected, to decide which simulators’ variables should be read first and transferred to the other simulator. The processes happening in the simulator running the model with the faster timescale, 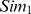, always needs to have up-to-date values at the beginning of each iteration loop. Therefore, the simulator from which the variables should be first read is the one dealing with the slower timescale 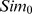. Indeed, (i) the simulator with the faster timescale is the one receiving external input, e.g. it is the one directly affected by the event; (ii) the simulator with the slower timescale will not be affected as soon as the event arrives, however the effect will be important after a relative long 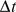, during which the behavior of the fast simulator 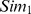 will have been strongly affected.

### Synchronization

**Figure 1:**
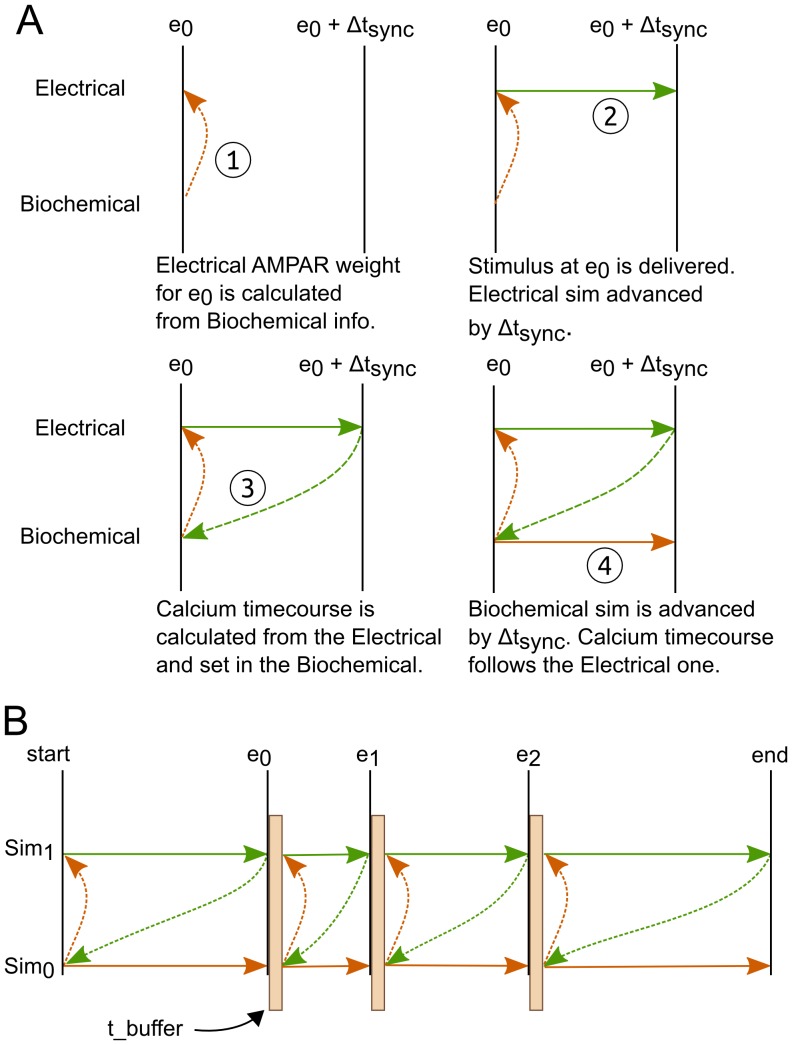
Synchronization principle. The dashed arrows refer to the variable exchanges from one simulator to the other. The solid arrows represent the time progression of the simulators. A, one synchronization loop. 1,2,3,4 represent the successive phases which are taking place at every synchronization cycle. B, repetition of the synchronization through several events. The brown boxes represent the synchronizations happening during one synchronization cycle. The duration of a synchronization is decided by the 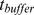 parameter. 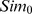 is the slow timescales simulator, 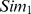 is the fast timescales simulator.

Figure 1a represents schematically how the two simulators are synchronized at timepoint of event zero (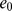). The synchronization involves four different steps, which comprise the exchange of corresponding variables between the simulators and the time increment. To synchronize the simulations at the time of event 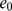, the relevant variables are read from 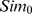, transformed and set in 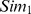 (1). 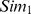 is then advanced by 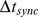 (2). At which point the relevant variables are read from 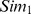, transformed and set in 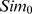 (3). 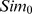 is then advanced by the same time as 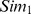 (4). Figure 1b represents a succession of events, until both simulators reach the end of the simulation.

**Figure 2:**
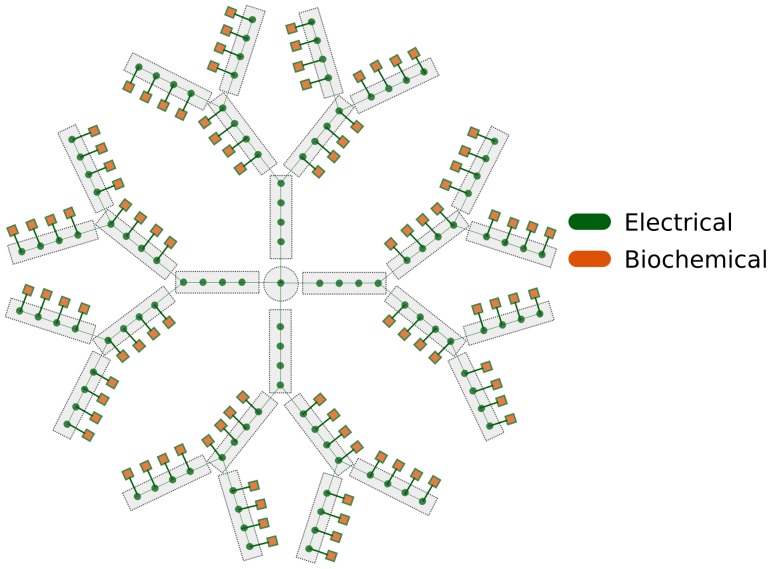
Schematic representation of the Hybrid Model. The electric model is shown in blue and the biochemical model is shown in red. Each spine’s biochemical model is connected with the spine’s electrical counterpart, all of which are integrated with the main electrical model of the neuron.

**Figure 3:**
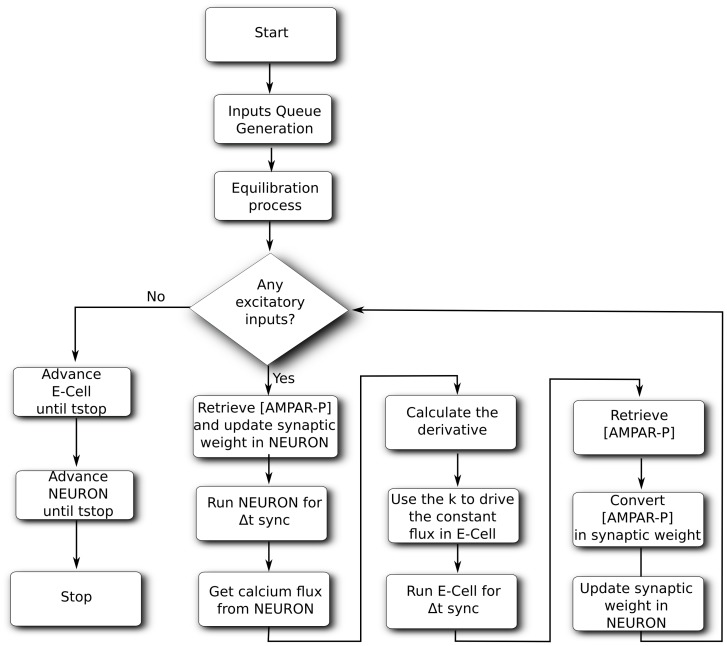
Event-driven algorithm applied to the Hybrid Model. Algorithm applied using E-CELL3 as the biochemical simulator (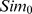) and NEURON as electrical simulator (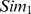).

The Hybrid Model of the MSN consists of an electrical model and a biochemical model connected together. Every spine has a double model, electrical and biochemical, which are connected and interact (Figure 2). During the synchronization loop, the relevant biochemical variables of interest are retrieved, scaled, converted into the corresponding variables of the electrical model. The electrical simulator is then advanced for a 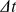. The newly computed relevant electrical variables are retrieved, scaled, converted into the corresponding variables of the biochemical model. The biochemical simulator is then run for the same 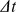. The synchronization loop is triggered each time an event happens. When all the events have been cleared, both simulators are advanced independently until 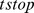 is reached. The flow chart of the event-driven algorithm applied to the Hybrid Model of the MSN is shown in Figure 3.

The synchronization event for the Hybrid Model is the release of Glutamate (Glu) at the level of the cortico-striatal synapse. It is modeled as trains of inputs of varied frequencies stimulating the AMPA and NMDA receptors present in each spine’s PSD section (see Method section). The times 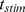, at which each input will be delivered, are computed before the simulation is started, and they are stored in a list. Both simulators are advanced until the first 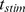 point. The synchronization loop is triggered and the current weight for the AMPAR synapse is calculated from the relevant variables in the biochemical simulator (see Methods). The stimulus is then delivered with the updated weight and the NEURON simulator is advanced for a 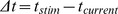. The same 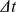 is used to advance E-CELL3. After the first synchronizations, the two simulators are forced to keep synchronizing with each other for a 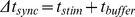, where 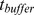 is an arbitrary time. During this 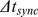, the two simulators are updated every millisecond, and the variables are exchanged between the two simulators. A detailed description of the variable exchange is presented in the Method section. After the synchronization loop has been completed, the synchronization loop is entered once again if there is another stimulus, otherwise both simulators are advanced until reaching the 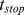. For example, if there is an event at time 1 s (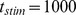) and 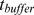 is set at 10 ms, 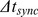, is equal to 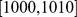 and during this delta the two simulators are kept in sync every ms. When the time 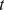 of the simulation is greater than 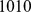 the algorithm returns to the fast regime and advances the two simulators separately until the next event or 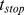.

### Comparing the Event-driven Algorithm with Other Synchronization Strategies

**Figure 4:**
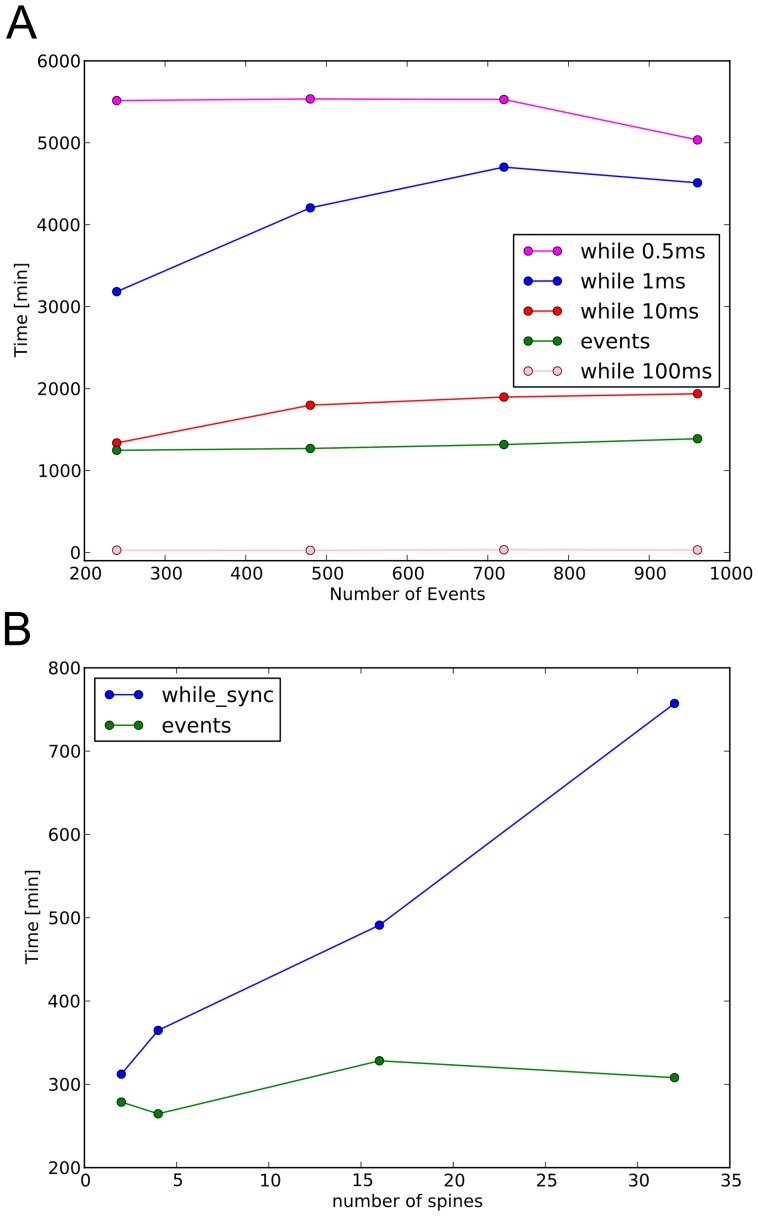
Comparison of the event driven algorithm with while loops and different sparseness. A, comparison of the event driven algorithm with while cycles. B, comparison of the event driven algorithm with while loops under different sparseness. The event driven algorithm offers a significant improvement over the usage of a while loop with a small 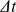. The slight improvement of the while loop with 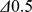 and 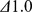 for the highest number of events is due to a different load on the cluster at the time the simulations were ran. B, scalability of the event algorithm with the increase of sparseness, compared to the while loop approach which cannot cope with it.

If the two simulators are synced at regular fixed timesteps with a while loop, the number of synchronization instances is determined by the duration of the stimulation, slowing down the computation. An event-driven algorithm reduces the number of synchronization events only to the stimulation events time, switching to a smaller 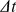 when a synchronization happens, and advancing the simulators separately until the next synchronization event, or the end of the simulation if there are no more events in the queue. To evaluate the framework, the same set of simulations have been run using the events-driven algorithm and a while cycle. The rollback method proposed by [23] has also been taken into consideration. However, it cannot be used to test the Hybrid Model, because the simulators need to be advanced in parallel, while in the Hybrid Model, the simulators must be advanced in precise sequential order, as explained above. By comparison, in a while cycle, the two simulators are synced every 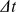, and run separately. Figure 4a shows the comparison between the event-driven algorithm and the simulations run using a while cycle, with different 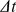. Table 1 shows how many stimulation events are missed by the while cycle. The events-driven algorithm is faster than the while cycle in the case the 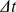 is very small. If 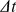 is larger the while cycle is faster at the price of missed events. It is important to stress that whereas intercepting the events is important, it is only one aspect of the synchronization. Another important aspect is how fast are the changes happening in the variables that need to be exchanged, and what is the best 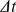 used to synchronize the two models. This value is intrinsic to the multiscale model. For example a value of 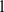 ms has to be used for the Hybrid Model to achieve an acceptable approximation of the transferred variables, as explained in the Method section.

**Table 1:** Missed events.

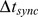	Total events	Events missed	Percent
10	240	0	0%
10	480	0	0%
10	720	0	0%
10	960	0	0%
100	240	120	50%
100	480	240	50%
100	720	360	50%
100	960	480	50%

Missed events using the while synchronization with 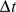 equal to 10 and 100 ms.

In the Hybrid Model, the sparseness of the model is defined as the number of spines stimulated in the simulation. Figure 4b shows how the event-driven algorithm compares with a while cycle under different sparseness. The computational time of the while cycle increases in a linear fashion with the sparse connectivity, from 2 to 32 spines stimulated. On the contrary, the simulation time does not depend on the sparseness in the case of event-driven synchronization.

### Single Spine Stimulation

To test the system, we ran a set of simulations with all the spines instantiated in the electrical model, and only one stimulated directly. The spine was stimulated with two different conditions: (i) a two pulses train with an interval of 100 ms and (ii) a train at 8 Hz for 2500 ms, i.e. 20 pulses with an interval of 125 ms. In the first condition, the first train was applied at 230 and 330 ms, while the second train was applied at 15100 and 15200 ms (Figure S1). The calcium which entered the spine according to the electrical model was converted and injected in the biochemical model. This amount was not sufficient to trigger the binding of Calmodulin to CaMKII, and activate the phosphorylation of AMPARs (Figure S2). A completely different response was obtained in the second condition, with two interesting results emerging (Figure S3). The first stimulation train starts at 2230 ms, while the second one starts at 15100 ms. The first train is able to trigger a higher depolarization of the spine, due to the increased weight (phosphorylated AMPARs, Figure S3b). This depolarization was clearer with the second input train, when the weight is increased by the previous *stimulus*, even after a delay of 15000 ms. Increased weights result from the activated Calmodulin-CaMKII complex able to phosphorylate AMPARs, increasing their number in the PSD (Figure S4), and therefore the weight of the synapse. The number of phosphorylated AMPARs kept increasing in the absence of stimulation because of the slower timescales of the biochemical reactions. When the second train arrives, the weight of the synapse has started to decrease due to the action of PP1. However, it it still higher than during the first train, resulting in a stronger depolarization of the spine, which also propagates to the soma level (Figure S3c).

We compared the effect of the biochemically-regulated synapses of the hybrid model with an electrical-only model of the spine. The stimulation of the spine with two successive trains reveals that the second train is able to elicit a stronger response than the first. The increase is proportional to the frequency of stimulation. The cause of the increased response is the increased weight of the synapse, mirroring the increased number of AMPARs produced by the biochemical model. With the biochemical model turned off, no differences can be observed between the responses elicited by both trains, as shown in Figure S5.

The ability of the spine to respond in different manners to two stimuli is well known [16]. What this model allows is the possibility to investigate how the spines can interact with each other at different times, when the stronger response is made available via biochemical memory, which would be completely lost if only an electrical model was used.

### Multiple Spine Stimulation

In the introduction, we have presented the CPH and how different spines can influence neighboring spines. In particular, Govindarajan et al. [13] have suggested that spines are working in engrams. When one spine gets stimulated, the plasticity of the neighboring ones is influenced, although indirectly.

**Figure 5:**
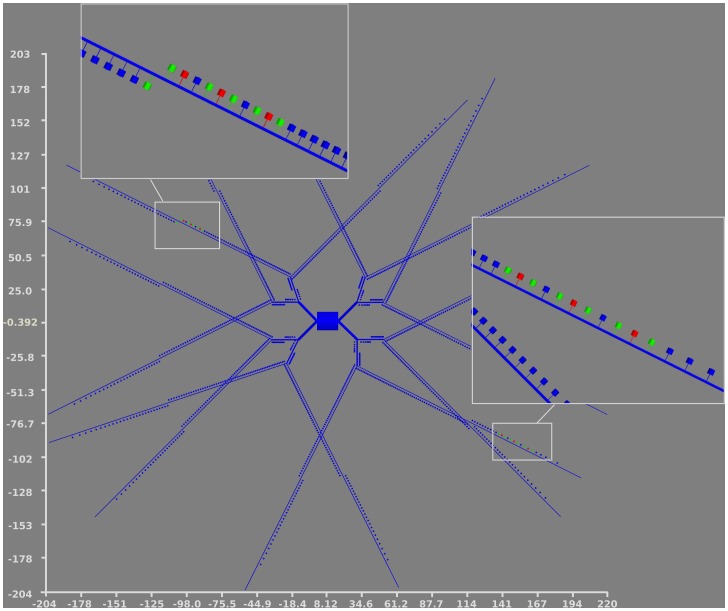
Spines stimulated by the first and second trains of input. Upper left, the “500” series; bottom right the “1400” series. The red spines are the ones receiving the double trains, the green ones are the ones receiving only one train. The axes are in 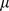
m.

To understand how neighboring spines could influence each other,we have stimulated 18 spines: nine spines (554, 555, 556, 558, 559, 560, 562, 563, 564) located on the distal dendrite dend1_1_2 and another set of nine spines (1468, 1469, 1470, 1472, 1473, 1474, 1476, 1477, 1478) on the symmetric distal dendrite dend4_1_2. The stimulation consists of two trains delivered at different frequencies: 8, 20, 40 Hz, each one of 50 inputs. A fourth stimulation at 40 Hz of double duration was also performed. The first train is delivered to all the spines while a second train is applied only to the spines 555, 559, 563, 1469, 1476, 1473, as shown in Figure 5. The number of stimuli, the frequency and the duration of each train are summarized in Table 2. The first train always starts at 2000 ms and the second train at 15000 ms.

**Table 2:** Characteristics of stimulation trains.

Frequencies	Inputs	Duration
8 Hz	50	6.25 s
20 Hz	50	2.50 s
40 Hz	50	1.25 s
40 Hz long	100	2.50 s

Durations, frequencies and number of events for a single train of stimulations.

No action potential was triggered at the soma level. At the spine level instead, a consistent depolarization was achieved, both on the spine directly stimulated and on the neighboring spines, not directly stimulated during the second train of inputs. This result is possible because while the first train is delivered to 18 spines, the second train stimulates only 6 of them. Their response is increased by the plasticity mechanism operating at the biochemical level, increasing the number of phosphorylated AMPARs and therefore the synaptic weight in the electrical synapse.

### Effect of Frequencies

**Figure 6:**
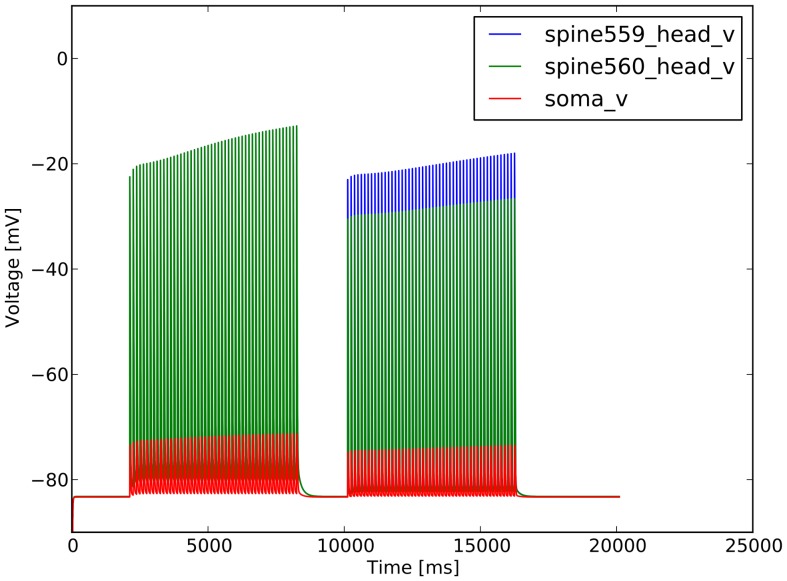
Response of the spine to the first and second trains. Spine 559 is stimulated with both trains, spine 560 only with the first one.

**Figure 7:**
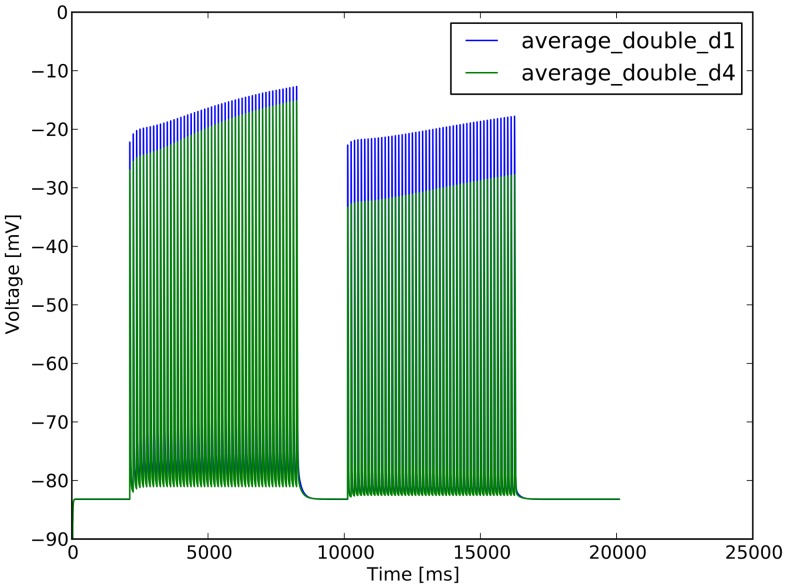
Different responses of spines differentially located. d1 is the average for the spines of the “500” series, closer to the soma, in dendrite dend1_1_2, while d4 is the average for the spines of “1400”, farther from the soma, in dendrite dend4_1_2.

Figure 6 shows how the spines react to the 8 Hz stimulation; the depolarization is high in the spines, but influences only marginally the soma. At the arrival of the second train, spine 560 gets depolarized with the current coming from spine 559 and the contribution from the other 2 spines in the same dendrite, 555 and 563. Spine 559 is used as the example of the double stimulated spine, while the 560 is plotted as an example of a singly stimulated one. The difference of depolarization for the two sets is due to the different axial resistance of the dendrite, which diameter decreases in function of the distance from the soma. The spines stimulated on dendrite dend1_1_2, 500 series were in the range 
m from the soma, while spines on the dend4_1_2, 1400 series were in the range 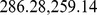
m. The spines closer to the soma are attached to a larger dendrite, thereby they experience a smaller axial resistance. This permits to achieve an higher depolarization (see Figure 7), which in turn influences the biochemical system, and therefore the synaptic weight.

**Figure 8:**
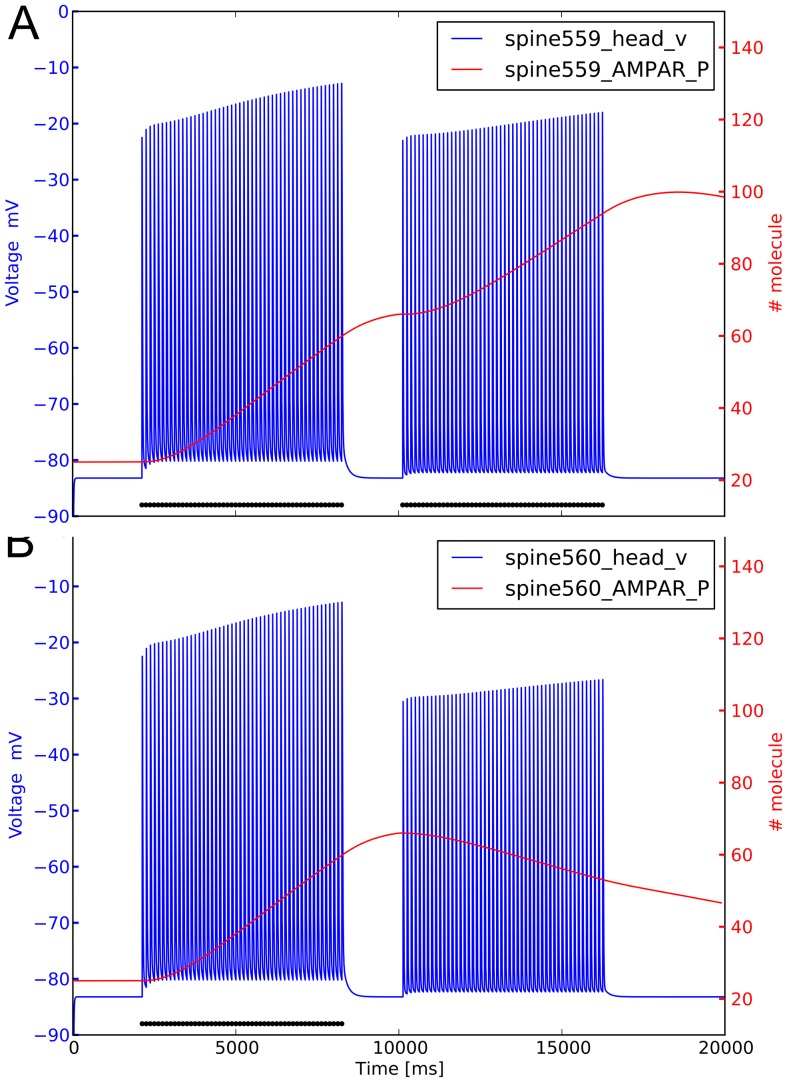
Depolarization and variation of phosphorylated AMPARs triggered by two trains of input. Spine 559 receives two trains of stimuli, while spine 560 receives only one train. A, spine 559. B, spine 560.

To analyze in detail the effect of electrical stimulations on biochemical pathways, we focused on spines 559 and 560. The former receives a double stimulation and the latter a single one. The timecourse of biochemical AMPARs is plotted in Figure 8 together with the electrical timecourse and the times of stimulation (black dots). During the first stimulation, the weight of the synapses carried by both spines increases after the first train, peaking around 10000 ms. The behavior is completely different when the second train is delivered, with an increase in the direct stimulated spine 559 and a decrease in spine 560. Calcium entering the spine followed the same kind of timecourse, increasing twice in the doubly stimulated spine 559. The difference is due to the contribution of the increased number of AMPARs, which are permeable to calcium. This also causes a positive feedback through the increase of active CaMKII, and the recruitment of phosphorylated AMPARs to the PSD. The situation is different for spine 560 where the number of AMPARs continues to increase after the first train, peaking around 10000 ms. In this model, calcium diffusion from one spine to another is not explicitly modeled, therefore the small amount of calcium entering the spine 560 is the result of the depolarization induced by the neighboring stimulated spines, especially via the contribution of voltage gated calcium channels (VGCCs) (Figure S6).

In the 20 Hz simulation, which features only 50 inputs but runs for 2.50 seconds instead of 6.25 seconds in the 8 Hz, the synaptic weight increases very quickly with the first train, reaching the peak around 8000 ms. The second train hits the spines at 15000 ms, causing a depolarization in both spines. In this case, the number of AMPARs phosphorylated by CaMKII is higher than in the 8 Hz stimulation. Moreover, when the second train is delivered to the spines, this number is already decreasing, with the consequent decrease of the synaptic weight.

**Figure 9:**
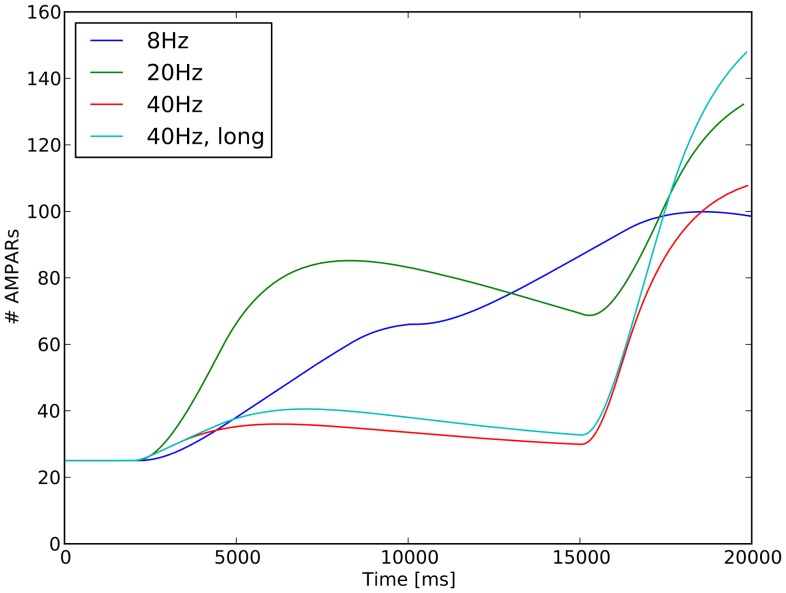
Effect of stimulation frequency on AMPARs phosphorylation in the stimulated spine. The same amount of inputs are delivered for all the frequencies but *40 Hz long*. The lower frequencies are able to trigger a higher phosphorylation, and therefore a higher conductance of the AMPARs in response to the first train. However, in response to the second train the high frequencies can still trigger a comparable phosphorylation of the AMPARs, even if the inputs are delivered after a large amount of time due to the stiffness of the biochemical pathways.

The model was also stimulated at 40 Hz with two different lengths of trains: (i) 50 inputs for 1.25 seconds (Figure S7), (ii) 100 inputs for a total length of 2.50 seconds (Figure S8). In none of the cases, the number of phosphorylated AMPARs during the first train increases as fast as in the 20 Hz stimulation. But it massively increases on delivery of the second train. Figure 9 summarizes the biochemical response triggered by the different frequencies. All the stimuli deliver the same amount of inputs, except for the long train at 40 Hz, which delivers twice as much, as described in Table 2. The ability of the slow frequencies to trigger a larger response on the first train is due to the distribution of the inputs across a longer time, which gives the biochemical system time to respond according to its own timescale. Therefore, as far as the biochemical system goes, the distribution of the initial inputs has already a major impact. When the second train arrives, the key for the faster phosphorylation of the AMPARs lies in the activity state of the biochemical memory. For the 40 Hz stimulation, it allows the system to provide a response of the same magnitude as for the 8 Hz and 20 Hz stimulations.

**Figure 10:**
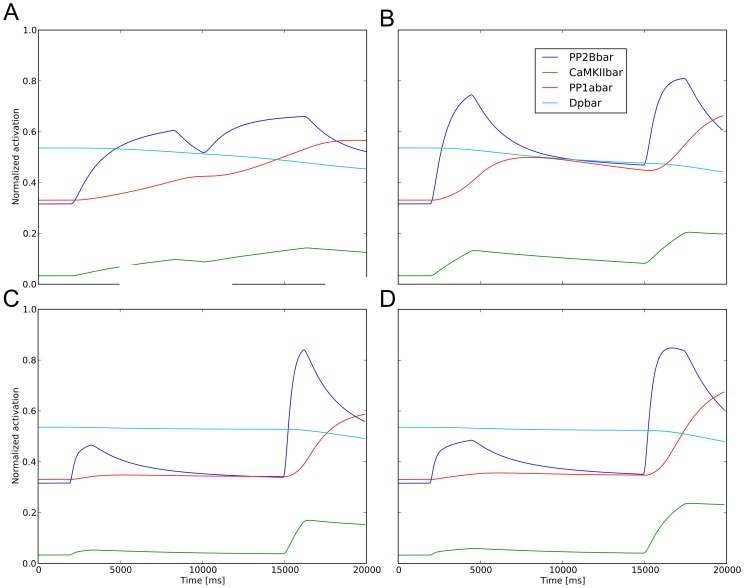
Fractional activation of enzymes for different stimulations. All curves correspond to spine 559. A, 8 Hz; B, 20 Hz; C, 40 Hz; D, 40 Hz long stimulation. The long intervals between successive entries of calcium in the 8 Hz and 20 Hz stimulations allow CaMKII and PP1 to get activated (after calcium binds calmodulin). The number of phosphorylated AMPARs increases because CaMKII concentration is higher than PP1 concentration. The situation is different with the 40 Hz stimulation, where the first train is too short to activate CaMKII significantly, causing only a small increase. However, when the second train arrives, CaMKII is activated quicker than PP1, causing an increase of phosphorylated AMPARs.

The different behaviors produced by the four stimulations are directly related to the evolution of the biochemical system, and the subsequent synaptic weight determined by the phosphorylation and dephosphorylation of AMPARs by CaMKII and PP1 respectively. In the absence of stimulation, the number of phosphorylated AMPARs is kept at equilibrium by the CaMKII and PP1. The action of PP1 is inhibited by the phosphorylated form of DARPP-32, which is under the control of PP2B (see methods). An overview of the timecourses for the biochemical system is plotted in Figure 10. For the 8 Hz stimulation, the flux of calcium is spread over a long interval, which permits both PP1 and CaMKII to be slowly activated (Figure 10a); CaMKII is present at the spine at a higher concentration than PP1, and therefore able to phosphorylate more AMPARs. At 20 Hz, Calcineurin dephosphorylates DARPP-32 (Figure 10b). DARPP-32 is therefore not able to inhibit PP1 any longer and at the same time the concentration of active CaMKII is decreasing. This delay introduced by Calcineurin and DARPP-32 explains the peak of the AMPARs visible in Figure S9 around 8000 ms. The situation is different for the 40 Hz stimulations. In both short and long stimulations, the first train is not able to cause a consistent increase of CaMKII, because it is too brief (see Figure 10c and Figure 10d). However, when the second train arrives at 10000 ms, CaMKII is activated very quickly, comparatively to PP1, which gets activated by the Calcineurin-DARPP-32 pathway and has an effect at a later stage.

### Contribution to the Plasticity of Neighboring Spines

**Figure 11:**
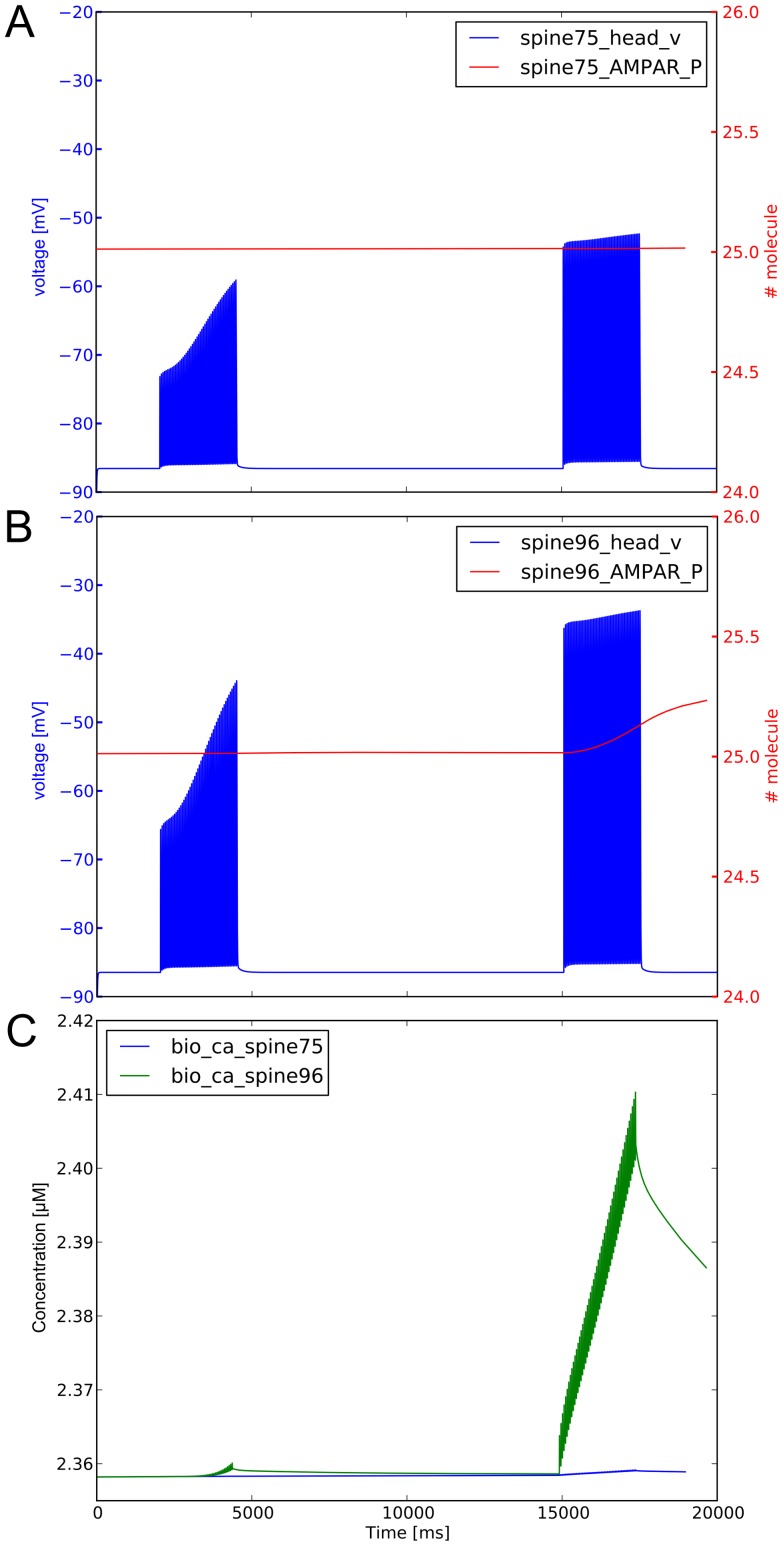
Response of non stimulated spines. Influence of stimulation of spine 97 on depolarization and number of AMPARs of distant spine 75 (panel A) and neighbor spine 96 (panel B). The amount of biochemical calcium in the two spines is plotted on panel C.

Govindara et al. [9] have stimulated spines on an hippocampus dendrite and shown, by monitoring the increase of spine head’s volume, that the synaptic weight of neighbors of stimulated spines was increased. We performed a similar simulation where only one spine was stimulated with a double train at 8 Hz, and the responses of the closest spines, together with a spine distant *circa* 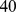
m were tracked (see Figure S10). To decrease the computational time, only the spines on the same stimulated dendrite was instantiated. Figure 11 compares neighbor and distant spine. As expected, the depolarization of the distant spine 75 (Figure 11a) is less pronounced than the one of the neighbor spine 96 (Figure 11b). On the arrival of the second input, the depolarization is stronger due to the presence of more AMPARs in the stimulated spine. The higher depolarization is able to open VGCCs, causing a subsequent increased amount of calcium to enter the biochemical model, (Figure 11c). This result is consistent with the result found by Govindara et al. [9].

## Discussion

The plasticity of a synapse carried by a spine results from complex interactions between biochemical networks and electrical channels [7]. These interactions are usually not modeled, the modeling effort focusing either only on the biochemical aspects or on the electrical ones. In the simulation we presented, two main questions have been addressed: (i) how different frequencies affect the response of a spine when the biochemical contribution is also taken in consideration, (ii) how a stimulated spine influences neighboring spines. While it is known that the input frequency and the duration have an effect on the depolarization of the spine [24], the role of the biochemical component is usually not investigated due to the longer timescales. We studied how the response of one spine stimulated with different trains of inputs changes, highlighting the role of the biochemical contribution on the increase of the synaptic response, and the resulting depolarization of the spine in response to the different stimulations. The overall effect contributes to substantial changes in the voltage response, not visible if the biochemical contribution is not taken into account (Figure S5).

### How Spines Influence each Other

The ability of spines to influence one another is a subject of much research, starting with the debated role of the neck [25]–[31] to the mechanism of inter-spines synaptic plasticity. The dispersed plasticity model suggested by Frey et al. [22] postulates a synaptic tag, that identifies spines in which the strength of the synapse should increase, and those in which it should decrease or even disappear. The synaptic plasticity would therefore result from a process happening solely within the stimulated spine. On the contrary, the clustered plasticity hypothesis proposes that spines work together as an ensemble and influence each other, as advanced by Govindarajan et al. [13]. The influence of a stimulated spine on the plasticity of the surrounding ones was shown by the same authors later [9].

Two main aspects can be highlighted in our simulations: (i) the biochemical component of the response of the stimulated spine to the first input train increases the electrophysiological response to the second input train, which in turn contributes to a larger depolarization of the surrounding spines. This triggers the opening of the VGCCs, resulting in an increased flux of calcium in the biochemical model (directly derived from the electrical one as explained in the Method section); (ii) the smaller response of the distant spines is not sufficient to increase calcium flux enough, and does not affect visibly the synaptic plasticity, in contrast with the closest spines (Figure 11). We conclude that the amount of calcium, the position of the spine on the dendritic tree, the location relative to the other stimulated spines, the timing of the stimulus, and the state of the biochemical enzymatic cascades, are all connected variables which influence each other in a complex manner.

Multiscale modeling is an approach used to address complex phenomena, which variables span several orders of magnitude. For this reason, it is used in several scientific fields, such as climate and weather forecast [33] and approaches used to integrate molecular dynamics and quantum physics [34]. Examples can also be found in biology, such as carcinogenesis and physiology [35]. To create a multiscale model, it is possible to combine submodels of interest with the use of analytical techniques or by creating an interface between them. Analytical methods can only be used when the models to combine are simple [1], while different types of interfaces can be used in the case of complex submodels [36]. In a multiscale model built with two interlinked modules, the timing of synchronization is a major factor to take in account, together with the number of times the synchronization happens. Borrowing the terminology from the study of networks, it is possible to define the interlink as connectivity, which could be defined as *sparse* when the number of synchronization requested during the whole simulation is low, and *dense* when the number of synchronization between the two models is high.

In a dense connectivity scenario, one solution is to synchronize the modules at a fixed 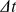 throughout the entire simulation. The size of the interval is critical. A small interval will ensure an accurate synchronization between the systems, however it will be computationally expensive. Conversely, a large interval will speed up the simulation, but the likelihood to miss a synchronization event will increase. This approach has been used by Ray et al. [37], with a one millisecond as fixed interval, in one of the first works to integrate electrical and biochemical signaling. In a recent paper, [38] have presented a model of a whole *Mycoplasma genitalium* cell, integrating 28 different modules, coupled through 16 global variables. The authors assumed that the modules were independent on a short timescale. They therefore decided to synchronize all the modules every second.

In a sparse connectivity scenario, if the two models are simulated in a parallel fashion, a rollback technique can be used, as demonstrated by Cvijovic et al. [23]. Although the work has been carried out on yeast, the technique is of interest in synchronizing any models. The rollback technique consists in advancing the models as far as possible in time, while an event detection system makes sure there is no need to synchronize them. If an event happens, and a synchronization is necessary, the models are rolled back to the latest time point before the event happened. They are then synchronized and the simulation continues, again running them separately as far as possible. The rollback action is expensive, therefore this technique can be applied only if the connectivity is sparse and if the models models are running in parallel.

A different solution to the problem of synchronization has been proposed by the Multi Simulator Coordinator (MUSIC) [39], a library which facilitates the interaction between simulators, taking advantage of the Message Passing Interface (MPI) [21], [40] for the communication. MUSIC does not take into account the sparseness of the connectivity. Instead it defers the decision to simulators, providing an Application Programming Interface (API) to make them synchronize. The layer between the models simulated by different simulators can be implemented with the MUSIC library [39], which permits a one way stream of data from one simulator to another. MUSIC has been used as the communication tool between a detailed population of neurons modeled with PyMOOSE [37], and a bigger population of artificial neurons simulated using NEST [41] in 2010 [42]. The variable shared between the simulators was the spikes’ time, which resolves into a stream of floats passed between the simulators. To achieve the synchronization, every simulator which supports MUSIC has the ability to emit a *tick*, a signal meaning the possibility to share the internal variable at that time point. Every *tick* is therefore used as checkpoint, where the variables are exchanged. The synchronization of this point is achieved using a while loop, and the *ticks* need to be emitted at regular time. As the *dt* for the numerical integration gets smaller, the number of *ticks* required increases [39]. This approach is efficient only when the connectivity between the two systems is dense, justifying a constant while loop to sync the simulators. However, in case of sparse connectivity, the constant synchronization between the two systems creates an overhead.

A sparse connectivity takes place when we consider electrical and biochemical simulations together. One important difference between a biochemical and an electric process lies in the time needed by each process to produce a change in the system. For example, a synaptic input triggers a voltage response in a spine head which peaks in 100 ms, while the phosphorylation of AMPARs takes minutes to peak. A multiscale approach has been used to run coupled simulations of biochemical and electrical activities by [37], where the two systems are run separately and then synchronized with a while loop at a constant *dt*. The variables are copied from one system to the other, and the simulation is then run until completion. The time at which the simulators need to synchronize is hard-coded in the model, without being driven by events. In our case, the time of the events/stimuli is set at the beginning of the simulation according to which protocol is used. It is not hard-coded in the algorithm, which make re-using the same algorithm easier with any set of events.

A consistent strategy, usable with both sparse and dense connectivities and that minimizes the number of synchronization between the modules would help advancing multiscale modeling in computational neuroscience. In this paper, we present a possible solution to the problem, with an event-driven algorithm which can be used in conditions of sparse and dense connectivities and which limits the number of synchronizations. The algorithm advances the simulators separately for as long as possible, and synchronize them only at the event time, guaranteeing the time consistency at the synchronization step. This brings two main results: (i) no unnecessary synchronization is performed, (ii) the simulators are synchronized at a precise time. The internal numerical integration strategies used are therefore completely decoupled from the synchronization algorithm itself. Another advantage of the event-driven algorithm is that simulators do not have to implement *ad hoc* support, but only exposes three public methods which usually are already used privately, or can be easily implemented. The algorithm is able to decrease the computational time when the connectivity is sparse, and provides a flexible way to address several inputs at different times. It has been tested with a set of events known before the simulation, such as a train of inputs at a certain frequency, This does not allow variable events emerging from the simulation. A way to overcome this limitation is presented in the Methods section, where the event driven algorithm is extended to accept events created during the simulation. The algorithm is integrated in the TimeScales framework, which uses Python as its main language and tested with the simulators NEURON [43] and E-CELL3 [44].

The way variables are exchanged at the level of the interfaces can have an impact on the results of the multiscale model. The approach used by Ray et al. [37] is to copy the current variable of interest, e.g calcium concentration, from the electrical simulator directly into the biochemical simulator. This strategy can be adopted when the variable exchanged is not subject to a quick change in the biochemical simulator. If the variable changes very quickly in the biochemical simulator, an instantaneous increase of the value could lead to artifacts, where the value could jump between high and low. For example, in the biochemical model of our Hybrid Model, calcium is quickly buffered by Calmodulin. If an inject type strategy is used, the concentration of free Calcium will change rapidly between a high value after the value is copied, and a low value as soon the Calmodulin buffers it. This creates an artifact, where the rise and decline of the free Calcium in the electrical model is not mirrored by a rise and decline in the biochemical model. We propose a solution which uses the first derivative of the variable free Calcium in the electrical model to calculate the constant for the flux of Calcium entering the biochemical model. The flux in E-Cell is implemented as Unimolecular Flux which provides a timecourse of calcium concentration in the biochemical simulator resembling the electrical one. The current solution calculates the constant to drive the UniMolecular Flux as the derivative of the calcium concentration at time 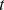 and 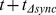. This offers a good estimation. One possible way to improve this approach would be take into account the slope of the electrical calcium concentration in order to compute a variable 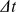 used in the synchronization phase.

### Interaction between Electrical and Biochemical Components in the Hybrid Model

Electrical and biochemical processes are tightly coupled in determining neuron properties and behaviors. However, until recently they have rarely been modeled together. The reasons behing such a division are diverse, including the difference of communities, the different mathematical frameworks, and the different timescales involved. Nevertheless, the biochemical cascades are influenced by the opening of electrical channels, whose states are in turn influenced by the biochemical cascades. Examples of this interaction loop are the influence of the MAPK pathway on the AMPARs [45], the integration between potassium channel activity and the dopamine controled cascades [46], and the effect of DARPP-32 as a key switch responding to dopamine and glutamate signals [47].

To integrate the electrical and biochemical aspects of the signaling in MSNs, we modeled the calcium-modulated pathway Calmodulin-CaMKII-Calcineurin, which influences the weight of the synapse by changing the number of phosphorylated AMPARs in the post-synaptic density. To model the feedback loop, we developed the Hybrid Model of the MSN, which is the first multiscale model of the MSN to date. The Hybrid Model features a symmetric dendritic tree with electrical conductances distributed according to experimental data [48], together with 1504 explicitly modeled spines, each one containing an electrical and a biochemical component, see Methods section.

We showed that 1) modeling the biochemical aspect of the synaptic signaling has a major impact on the electrical response of the spine, providing a plasticity rule for the increase of the synaptic weight, compatible with the low resistivity of the neck as suggested by [30]; 2) stimulated spines are able to influence the plasticity of neighboring spines, forming an engram as suggested by [13], without affecting the plasticity on distant spines. These two results are emerging from the current version of the Hybrid Model, which takes into account only one biochemical cascade known to influence synaptic plasticity. Several other signaling pathways are involved in shaping the plasticity of the spines, some of which are significantly altered in diseases, like Parkinson’s [49], [50]. In particular, the role of dopamine as a neuromodulator, able to change the effect of glutamate [47], [51] could be investigated. DARPP-32, stimulated by cAMP and therefore activated also by dopamine, is actually already present in the biochemical model.

Since the Hybrid Model is run using the modular Timescale framework, it would be possible to integrate other simulators, like STEPS [2], to explore the contribution of three-dimensional diffusion of proteins such as Ras [18] to the plasticity of neighboring spines.

### Minimum Step to Create an Interface

Usually different models, dedicated to the study of a different aspects of life, are written for different simulators. Some work is therefore needed to solve the problems of communication and integration. Usually, any two simulators can be interfaced if both can offer at least three public methods: (i) step(), (ii) set(), (iii) get(). The method step() is used to advance the simulator to an arbitrary time point, while set() and get() are used to write and read the values of variables. All these methods should be callable at run-time, without breaking the internal integration method used by the simulator. Keeping those requirements in mind, we have explored possible solutions and suitable software to test them.

If both simulators are running as parallel applications using (MPI) [21], [40], it is possible to exchange variables and synchronize them using the MUSIC [39] library. At the time we started our project, the MUSIC library was not yet implemented in NEURON, or in any other simulation software able to solve biochemical models. The use of MPI does not offer any computational benefit for single cell models, therefore this solution was discarded.

Simulators are usually specialized to solve models using a given approach. This is in particular the case for electrical propagation of voltage using the cable equation, or biochemical cascades using chemical kinetics. GENESIS [52] with the KinetiKit extension [53] tried to merge both visions of the neuronal world, proposing a simulator able to solve biochemical signaling and electrical modeling. The way to connect the two parts of the simulator is only stated in the book of GENESIS [52] as technically feasible. However, to the best of our knowledge, no working model has been published. GENESIS core was rewritten in the Multiscale Object-Oriented Simulation environment (MOOSE) and in 2008 [37] presented a way to couple NEURON with PyMOOSE, the Python binding of MOOSE. While this was the first attempt to run electrical and biochemical simulation together, the integration between the two systems was achieved with a loop synchronizing with a fixed 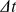 of 1 s. More recently [45] explored the multiscale modeling approach using solely the MOOSE software. The multiscale model consisted of 3 spines of an hippocampal neuron where biochemical processes were synchronized with electrophysiological ones using a while loop.

In our work, the electrical part of the multiscale model was modeled with NEURON [43], which provides a way to implement the three methods described above using a new Python interface [54]. For the biochemical component, different simulators were considered. We initially explored the COmplex PAthway SImulator (COPASI) [55] and SBMLOdeSolver [56]. However they did not expose the public methods we have identified above. We also tested the STochastic Engine for Pathways Simulation (STEPS) [2], a stochastic simulator which permits to model reaction diffusion in an arbitrary 3D space and the Python Simulator for Cellular Systems (PySCES) [57]. PySCES provides a Python runtime interface. However it was not possible to advance the simulation to an arbitrary point in time and continue the simulation from there. Therefore we have decided to work with E-CELL3 [44], which had the three public methods we required, and had a Python run-time interface. To achieve more flexibility, making sure the sync between two different simulators is performed only when it is needed, and to adapt to a complex firing pattern, we have developed an event-driven synchronization algorithm and fitted it into the *TimeScales* framework. TimeScales can be extended to incorporate any other simulators which offers a Python run-time control like PyMOOSE, STEPS or COPASI.

The work presented here can open up to more complex multiscale simulations, providing the ability to explore different combinations of modules. In particular, simulations could investigate the role of different biochemical modules which cannot be merged in one large model, combine an electrical module with the study of the dynamic morphing of morphology (for example in spine [29], [58], [59]), combine detailed multicompartment model of neurons and large scale network simulations [60], or explore the other end of the scale, combining a detailed multicompartment model with a thermodynamics energy module.

## Methods

### MSN Electrical Module

The dendritic tree described in [48] was used as a base for our electrical model. The symmetric tree is formed by 4 proximal dendrites, each dividing in 2 medial dendrites, subsequently dividing in two distal dendrites, for a total of 28 dendritic branches.

The fourteen electrical conductances distributed in the model are also based on [48]. They are divided in two sodium currents, Fast (NaF) and Persistent (NaP); six potassium channels, Potassium Inward Rectifier (K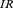, also known as Kv 2.1), Potassium slow A-type (K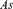, also known as Kv1.2), Potassium fast A-type (K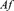, also known as Kv4.2), Potassium 4-AP resistant persistent (K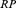), Potassium small conductance calcium dependent (SKK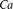) and Potassium large-conductance calcium dependent (BKK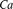); six calcium channels, N-type, Q-type, R-type, T-type, L-type Cav1.2 high voltage activated and L-type Cav1.3 low-voltage activated. The channels are modeled using the Hodgkin-Huxley formalism following the general equation 1, with 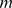 and 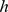 determined by equations 2 and 3 respectively.
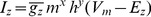
(1)

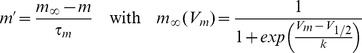
(2)

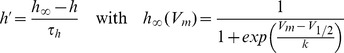
(3)



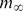
, 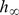, 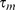 and 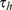 are the steady state activation and time constants for 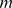 and 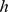 at voltage 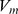. 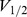 and 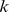 are the half-activation constant and the slope of the Boltzmann fit to 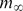 and 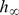 and 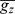 is the maximal conductance of the channel. The channels are implemented in NEURON as “mod” files. Four channels, K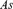, K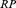, N-type and L-type calcium 1.2, are modeled using a modified version of the Hodgkin-Huxley equation, as described by eq. 4, where 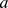 is a coefficient used to increase (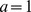) or decrease (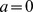) the inactivation of the variable.
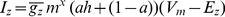
(4)


The parameters for the channels are taken from [48], and are reported in Table 3 for the potassium and sodium channels, and in Table 4 for the calcium channels.

**Table 3:** Parameters for the sodium and potassium channels.

	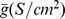	HHform		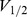	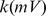	provenance of 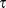
NaF	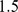 , soma	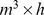	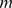	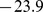	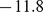	Tabulated
	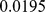 , dendrites		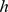	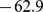	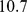	Tabulated
NaP	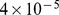 , soma	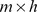	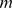	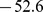	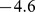	[48]
	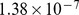 , dendrites		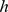	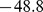	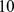	Tabulated
K_Af_	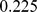 , soma and proximal	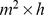	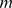	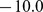	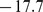	Tabulated
	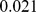 , middle and distal		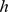	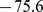	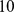	[48]
K_As_	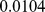 , soma and proximal	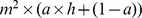	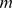	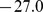	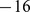	[48]
	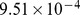 , middle and distal	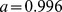	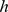	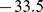	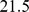	[48]
K_IR_	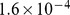	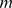	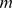	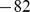	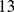	Tabulated
K_RP_	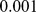	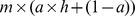	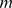	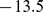	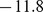	Tabulated
		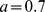	h	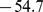	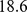	Tabulated
BKK_Ca_	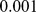					
SKK_Ca_	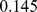					

The conductance of K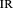 was changed from 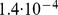 to 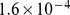 to match the membrane voltage when all the spines were instantiated.

**Table 4:** Parameters for the calcium channels.

	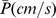	HHform		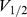	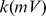	provenance of 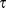
CaL1.2	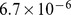	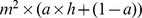	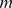	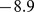	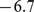	[48]
		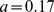	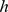	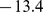	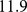	[48]
CaL1.3	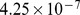	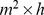	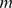	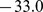	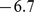	[48]
			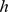	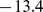	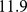	[48]
CaN	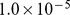	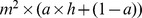	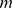	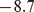	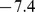	[48]
		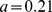	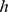	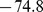	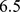	[48]
CaQ	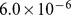	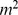	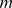	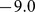	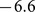	0.377 ms
CaR	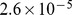	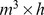	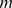	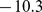	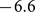	1.7 ms
			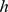	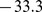	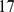	Tabulated
CaT	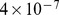	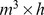	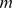	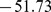	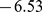	Tabulated
			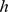	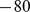	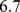	Tabulated

If the time constants had a fixed value, we used the value reported. In the case of dynamically calculated ones, we refer to the paper provided the mathematical expression used. In the case, the values are tabulated, they are calculated by NEURON at run-time, and their value can be found in the model code which is available (see below).

### Electrical Dendritic Spine Model

**Figure 12:**
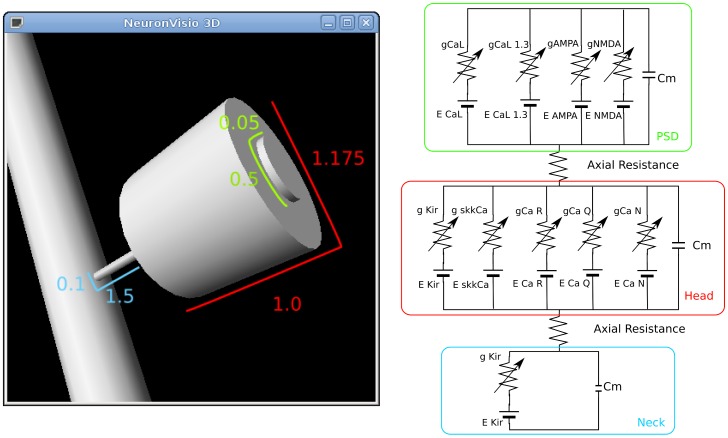
Spine dimensions and equivalent circuit. The dimensions are expressed in 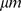, the post-synaptic density is in green, the head in red and the neck in blue.

The model of the spine is an extension of the one presented in [61]. Each electric spine is modeled using three electrical sections, PSD, head and neck, which are connected with an axial resistance as shown in Figure 12. Each spine is connected with the dendritic tree through the neck. The sections are modeled as cylinders with the geometrical dimensions shown in Table 5.

**Table 5:** Spine dimensions and surface.

Section	Diameter *(μm*)	Length (*μm*)	Surface (*μm* ^2^)
PSD	0.5	0.05	0.471
Head	1.175	1.0	5.86
Neck	0.1	1.5	0.487

The PSD is an internal section of the spine’s head and its contribution to the surface has not been included to the total spine’s surface, which measures 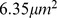. The channels inserted in the PSD are the L-type Cav1.2 high voltage activated and L-type Cav1.3 low-voltage activated [50]. The AMPA synapse and the NMDA synapse have been modeled as co-localized, as they have been shown to be in the CA1 hippocampal neurons [62], using a double exponential. The channels inserted in the spine head are the Q-type, R-type, N-type and SKK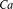. We have inserted the K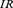 in the head and in the neck according to [46]. However, other potassium channels could be present instead of K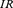, such as K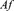 [49]. The spine has been developed as an *ad hoc* Python class, which initializes the three electrical sections and the biochemical simulator if an input is delivered.

### Spines Distribution

In [48], spines are not modeled explicitly, and a correction to account for the loss of total surface is applied, increasing the length and the diameter of the sections. It uses the Segev method [63], according to the equation 6, with 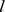 and 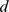 the length and the diameter of the dendrite respectively, and 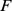 the fraction between the sum of dendrites and spines areas, divided the area of the dendrite.
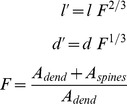
(5)


The total increased surface of the model is 14973 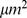, while the surface calculated with the original dimension is 5278 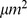. The number of spines to add to the model is 1526, obtained by dividing the difference, 9695 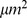, by the surface of the spine, 6.35 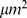. To keep the symmetry of the model, we reduced the number of spines to 1524, that is 381 per branch.

**Figure 13:**
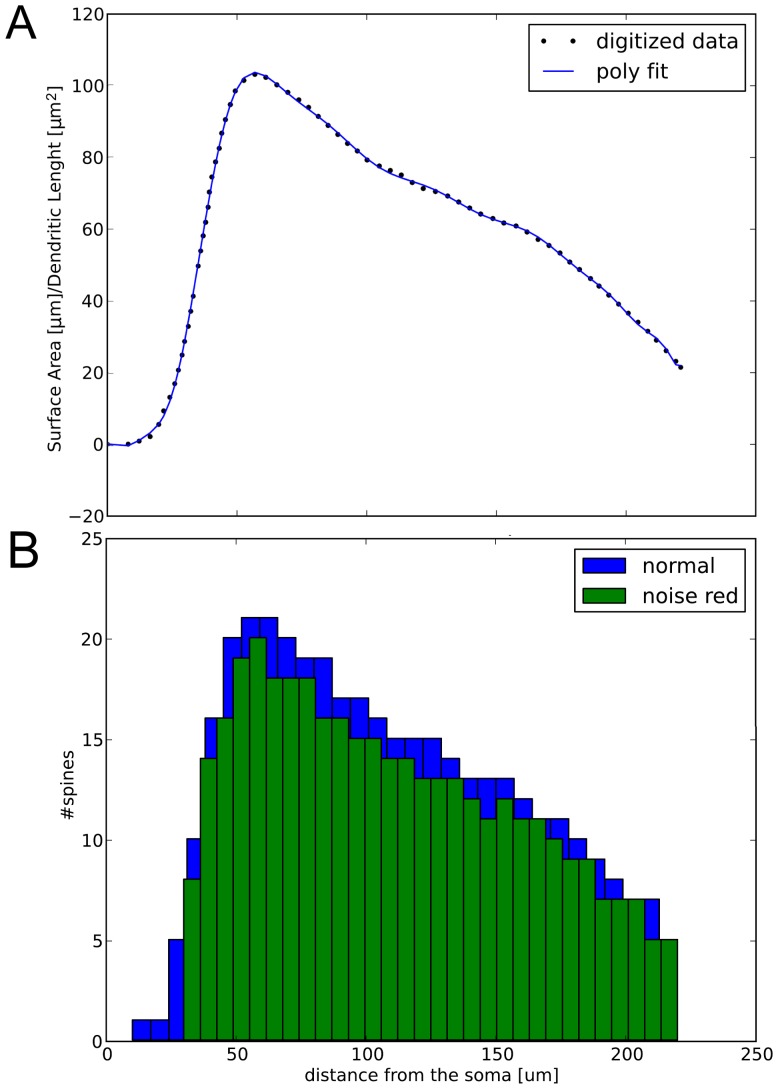
Fit of the spine membrane surface and spine distribution per branch. Panel A shows the polynomial fit (17^th^ order) of the spine membrane surface using digitized data from [64]. In panel B, the histogram of the spine distribution calculated with the equivalent spine surface is shown in blue. The final number of spines used (371 per branch, 1504 total), after removal of the noise due to spines positioned over the soma and the proximal dendrites, is shown in green.

The distribution of the spines is not uniform [64]. They are virtually absent on the proximal dendrites, very concentrated at the last part of the medial dendrites and the initial part of the distal dendrites (50 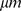 circa from the soma) and their density slowly decreases towards the end of the distal dendrites (see Figure S11). To calculate the distribution of the spines on the dendritic tree we digitalized the data from [64] describing the spine membrane surface in function of the distance from the soma. We then fitted the result with a 17^th^ order polynomial function, as shown in Figure 13b.

To obtain the total surface area of the spines for one of the four dendrites, formed by one proximal, two medial and four distal branches, we divided the total spine membrane area, obtained by integrating the polynomial, by four. The area has then been discretized using a spine equivalent area, to calculate how many spines should be inserted at each position of the dendrites. The result is the blue histogram, Figure 13b, which, due to the noise of the digitized data, shows a total of 5 spines at the soma and proximal dendrites’ location. These spines have been excluded, resulting in a new spine distribution, shown on the green histogram, with a total of 376 spines per branch.

### Biochemical Signaling Module

**Figure 14:**
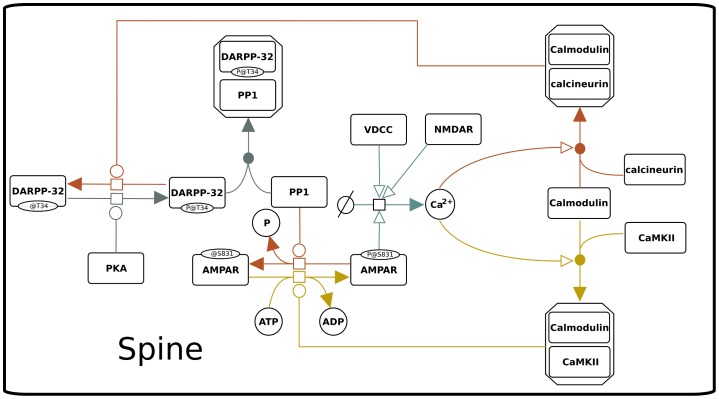
Interaction between ion channels and biochemical signaling. DARPP-32 forms a complex with PP1 after having been phosphorylated by PKA (grey line). Two possible pathways can be activated according to the concentration of calcium: at low calcium concentration Calmodulin forms a complex with Calcineurin, dephosphorylating DARPP-32, releasing PP1 inhibition, with subsequent dephosphorylation of AMPARs (orange line). At high calcium concentration, the complex CaMKII/Calmodulin is able to phosphorylate AMPARs (yellow line). The calcium flux incoming from the ionic channels AMPARs, NMDARs and VGCCs is represented in light blue.

The biochemical model, integrated with the electrical one, is represented in Figure 14 using the System Biology Graphical Notation (SBGN) [65]. The model includes the allosteric Calmodulin model from [66], the reactions between the Calcineurin-Calmodulin complex with DARPP-32, PKA and PP1 presented in [67]. Dephosphorylation of AMPAR by PP1 and phosphorylation of AMPAR by the complex Calmodulin-CaMKII were added for the purpose of the present work.

The source of biochemical calcium is the total flux of electrical calcium coming from the Voltage Gated Calcium Channels (VGCC)s, the AMPARs and the NMDARs, injected in the biochemical model after been scaled and computed as explained below. The amount of calcium influences the behavior of Calmodulin, an allosteric protein which can bind both Calcineurin and CaMKII [66]. When the amount of calcium is small, Calmodulin binds Calcineurin, forming a complex which is able to dephosphorylate threonin 34 of DARPP-32. DARPP-32 then no longer inhibits PP1, which is able to dephosphorylate AMPARs (orange lines in Figure 14). This in turn decreases the number of available AMPARs in the PSD. In the presence of high concentration of calcium, Calmodulin binds CaMKII and this complex is able to phosphorylate directly AMPARs on Serin 831, causing the incorporation of the protein in the PSD [68] (yellow lines in Figure 14).

Table 6 reports the parameters used for the allosteric model of the Calmodulin, taken from [66]. Table 7 reports the values and references for the constants used in the other reactions. Table 8 reports the initial concentrations.

**Table 6:** Calmodulin parameters for binding calcium.

Parameter	Value	Reference
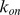	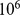 M^−1^s^−1^	[66]
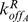	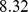 s^−1^	[66]
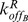	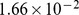 s^−1^	[66]
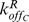	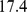 s^−1^	[66]
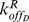	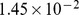 s^−1^	[66]
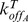	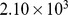 s^−1^	[66]
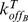	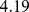 s^−1^	[66]
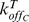	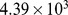 s^−1^	[66]
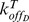	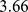 s^−1^	[66]
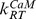	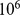 s^−1^	[66]
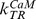	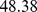 s^−1^	[66]
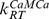	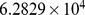 s^−1^	[66]
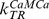	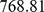 s^−1^	[66]
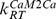	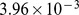 s^−1^	[66]
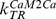	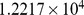 s^−1^	[66]
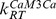	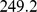 s^−1^	[66]
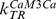	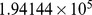 s^−1^	[66]
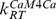	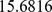 s^−1^	[66]
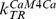	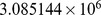 s^−1^	[66]

**Table 7:** Additional parameters for the biochemical model.

Parameter	Value	Reference
Ca2+ pump:		
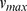	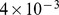 M s^−1^	[79]
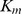	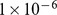 M	[79]
Ca2+ leak:		
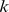	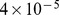 M s^−1^	[79]
CaMR binding targets:		
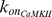	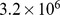 M^−1^ s^−1^	[79]
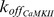	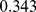 s^−1^	[80]
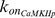	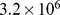 M^−1^ s^−1^	[80]
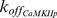	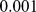 s^−1^	[80]
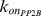	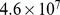 M^−1^ s^−1^	[81]
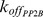	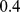 s^−1^	[82]
CaMKII autophosphorylation on Thr286:		
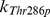	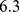 s^−1^	[83]
PKA phosphorylates DARPP-32 on Thr34:		
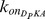	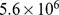 M^−1^ s^−1^	[84]
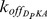	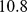 s^−1^	[85]
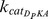	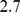 s^−1^	[85]
Calcineurin (PP2B) dephosphorylates DARPP-32 on Thr34:		
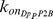	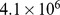 M^−1^ s^−1^	[85]
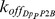	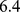 s^−1^	[86]
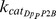	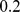 s^−1^	[86]
DARPP-32 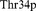 binding PP1:		
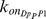	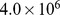 M^−1^ s^−1^	[86]
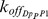	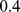 s^−1^	[87]
PP1 dephosphorylates CaMKII:		
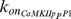	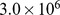 M^−1^ s^−1^	[87]
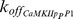	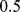 s^−1^	[88]
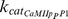	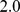 s^−1^	[88]
CaMKII phosphorylates AMPAR on Ser831:		
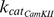	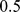 s^−1^	[89]
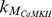	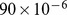 M	[68]
PP1 dephosphorylates AMPAR on Ser831:		
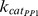	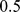 s^−1^	[68]
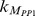	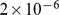 M	[68]

**Table 8:** Initial concentrations.

Concentrations	Value	Reference
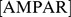	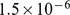 M	[90]
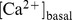	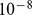 M	[91]
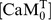	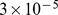 M	[66]
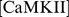	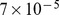 M	[92]
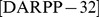	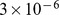 M	[88]
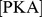	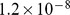 M	[93]
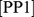	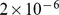 M	[94]
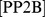	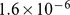 M	[95]

### Variables Exchange and Transformation

**Figure 15:**
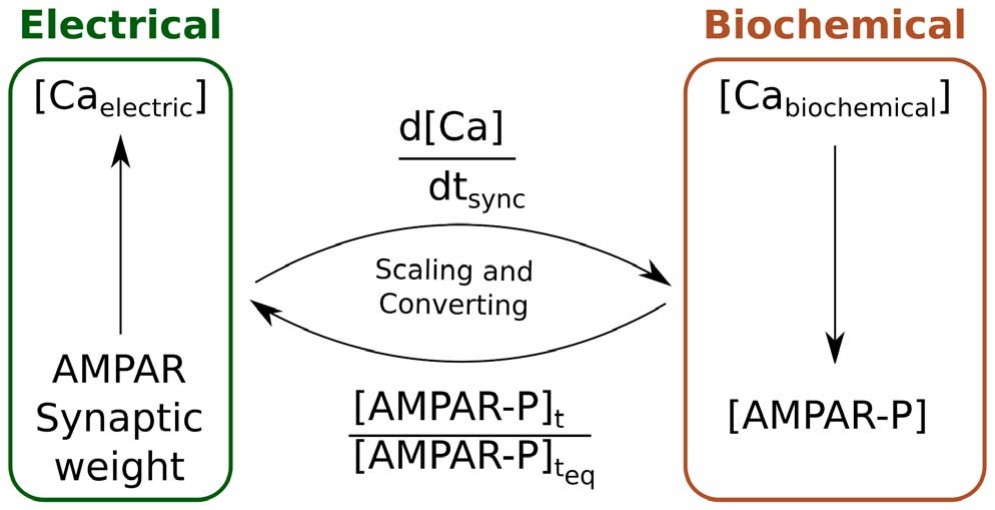
Feedback loop between calcium concentration and synaptic weight.

Synaptic strength is under the control of a feedback loop: the concentration of calcium in the spine after an excitatory stimulus is governed by the number and type of channels in the membrane, especially the VGCCs, which start to open only around -50 mV [69]. NMDARs and AMPARs permeable to calcium also contribute to the dynamics of calcium concentration in the spine [70]. One of the ways by which the weight of a synapse is increased or decreased consist in changing the number of AMPARs in the PSD [7], [8], [71]. The trafficking of AMPARs is a complex process which involves different pathways [68], [71]–[74]. AMPARs can be phosphorylated by CaMKII on Serine 831 and dephosphorylated by PP1 [68]. The phosphorylation increases the number of AMPARs in the membrane and therefore the flux of ions able to enter the neuron. In the Hybrid Model this feedback loop (Figure 15) is at the core of the interconnection between biochemical and electrical. The two models are updated during the synchronization cycle for a 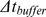 every 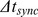.

In the electrical model, the intracellular calcium concentration in each spine is calculated as a thin shell around the membrane as in [48], according to the equation 6 [75],
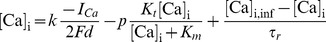
(6)where 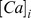 is the intracellular calcium concentration, 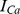 is the inward calcium current, 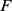 is the Faraday constant, equal to 96.489 C/mol, and 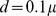
m is the shell depth. The pump term is represented using Michaelis-Menten formulation with a turnover 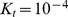 mM/ms and a Michaelis constant 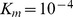 mM. 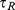 represents the diffusive term equal to 43 ms [76] and 
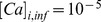
 mM as the equilibrium intracellular calcium. The parameters 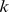 and 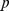 were left to their original values, 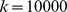 and 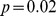, as in [48]. The biochemical concentration of the calcium is obtained by calculating the flux as the derivative of the concentration of the electric calcium during the 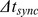, as shown in eq. 7.



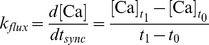
(7)

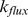
 is used to run for the same 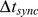 a ConstantFluxProcess, which is zeroth order reaction in E-CELL3. This process introduces the calcium in the spine. All the other biochemical reactions also take place at that time, with a possible change of the concentration of the phosphorylated AMPARs. The current approach to synchronize calcium from the electrical and biochemical models uses a fixed 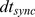. This solution could be improved, for instance using a variable 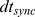 which follows the steepness of the electrical Calcium concentration. Figure S12 shows how biochemical calcium, composed of free and buffered calcium, approximates the shape of the electrical calcium.

Modifications of the weight of the AMPA synapses are usually expressed as the relative change between resting and excited conditions [70]. We used a similar approach to calculate the electric weight using the equation 8, where (#AMPAR-P)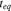 is the number of AMPARs at a time where the system is at equilibrium and (#AMPAR-P)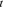 is the number of AMPARs at the current time 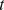. The result is normalized and used to change, at run-time, the weight field of the NetCon object for the respective synapse. The final synapse’s electric weight is a multiplicand of the total conductance of the AMPAR channel, and will affect the ion flux at the next stimulus in that particular spine.
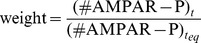
(8)


### Events Delivery and Biochemical Spines’ Relationship

Each spine can be individually stimulated with different spike trains. To deal with the complexity of the simulation, a workflow which involves the integration between Sumatra, Neuronvisio and TimeScales has been developed.

Sumatra [77] is a an electronic labbook for managing and tracking projects based on numerical simulations and analysis, with the aim of supporting reproducible research. It permits automated storing, tagging and retrieving of the results. Neuronvisio [78] is a 3D viewer for the NEURON simulator, which provides a modular system to save numerical arrays coming from different simulators in one coherent storage method, with the ability to reload the whole dataset at a later stage. While TimeScales is used to run the simulation itself and is responsible for the creation of the event queue and the instantiation of the required biochemical simulators, Sumatra is used to track which stimuli and parameters are used in each simulation, and Neuronvisio’s manager module is used to store the computational results.

At setup time, two actions have to be undertaken to ensure the events are delivered and processed at the proper time by each stimulated spine: (i) inputs must be instantiated in the electrical model at the proper synapse, (ii) a biochemical model must be created in the stimulated spines. The type of stimulation, frequency and duration, as well as the stimulated spines stimulated are defined in the parameter file, which follows Sumatra’s conventions (Sumatra uses the parameters module from NeuroTools package http://neuralensemble.org/trac/NeuroTools). In the electrical model the synaptic inputs are managed using a VecStim object that creates events to the specific synapses, i.e. NMDA or AMPA, following the vector, using the stimulus module in the neuronmanager package. Instead, the biochemical model is managed by the ecellManager object, which instantiates the biochemical model and brings it to the appropriate equilibrium. This approach allows to transform any existing electrical spine to one which has also a biochemical counterpart on demand, making sure the events are delivered to the proper synapses, and the spines will be synced before any inputs are delivered.

### Synchronization on Demand with Events Unknown Prior to Simulation

In case the events are not known beforehand, the algorithm can be adapted to perform the synchronization on demand when a new event is detected. To achieve this result, the fastest timescales simulator should be able to broadcast the event’s time. The fastest timescales simulator should always be advanced first. When an event is detected, the synchronization should be performed, calculating the 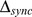 following the same procedure than when the events are known beforehand. The following listing explains how to achieve this, using NEURON as the fast simulator and E-Cell as the slow timescales simulator:

while (t<tstop):

 event_time = advance_neuron(tstep)

 advance_ecell(tstep)

 if event_time:

  synchronize(event_time)

### Model and Framework Source Code Availability

The model of the multiscale MSN, with the code of the TimeScales framework is available under the BSD license at https://github.com/mattions/TimeScales. The model of the multiscale Spiny Neuron has also been submitted to the Open Source Brain project http://www.opensourcebrain.org/projects/multiscale-medium-spiny-neuron-mattioni-and-le-novere. It will be uploaded to ModelDB.

## Supporting Information

Figure S1 **Two stimuli at different times on the same spine.** The two pulses are applied at 100 ms and 15000 ms; A, complete timecourses; B, Zoom on the first stimulus; C, zoom on the second stimulus.
(TIF)

Figure S2 **Effect of a short stimulation on the weight of the AMPA synapse.** A, weight applied to the synapse on the electrical model. B, phosphorylated AMPA timecourse which is used to calculate the weight. A small stimulus triggers a minimal variation of the phosphorylated AMPA.
(TIF)

Figure S3 **Single spine, response to a 8 Hz train stimulation.** Response of the MSN model to a 8 Hz train stimulation. The weight increases during the stimulation. The response to the second train is larger in the spine because of the change of the synaptic weight connected with the biochemical model, which changes the number of AMPARs. A, complete timecourses; B, responses to the first train starting at 2100 ms; C, responses to the second train starting at 10100 ms.
(TIF)

Figure S4 **Effect of a large stimulation on the weight of the AMPA synapse.** A, weight applied to the synapse on the electrical model. B, phosphorylated AMPA timecourse used to calculate the weight. A large stimulus triggers a significant variation of the phosphorylated AMPA, and the electrical response changes as a result.
(TIF)

Figure S5 **Difference of responses due to biochemical pathways.** Double stimulations of a single spine (532) with increased frequencies. Y-axis represents the difference between the average peak voltage of responses to first and second train of stimulations. In the hybrid model, the second train is able to trigger an increased response compared to the first one (blue plot). This is due to the increased weight of the synapse mirroring increased number of AMPARs produced by the biochemical model. Not surprisingly, with the biochemical model turned off, there are no differences between the responses elicited by both trains (green plot).
(TIF)

Figure S6 **Calcium response in the adjacent spines.** Calcium response of two adjacent spines. A, spine 559 receives two trains of stimuli. B, spine 560 only receives one train of stimuli. During the second train of stimuli, a small amount of calcium enters spine 560 due to the action of voltage-gated calcium channels activated by the stimulation of spine 559.
(TIF)

Figure S7 **Difference of AMPARs phosphorylation in adjacent spines following a 40 Hz stimulation.** A, spine 559 receives the first and the second trains of stimulation. B, spine 560 receives only the first train.
(TIF)

Figure S8 **Difference of AMPARs phosphorylation in adjacent spines following a longer 40 Hz stimulation.** A, spine 559 receives the first and the second trains of stimulation. B, spine 560 receives only the first train.
(TIF)

Figure S9 **Difference of AMPARs phosphorylation in adjacent spines following a 20 Hz stimulation.** A, spine 559 receives the first and the second trains of stimulation. B, spine 560 receives only the first train.
(TIF)

Figure S10
**Tracking the effect of biochemical depolarization on adjacent and distant spines.** Spines stimulated in one branch. Spine number 97, in red, is directly stimulated with two trains, while spines 75, 96 and 98, in green, are monitored to assess the influence of the electrical depolarization on the biochemical calcium. The axes are in 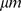.(TIF)

Figure S11 **The multiscale MSN model.** Multiscale MSN model rendered with Neuronvisio [78]. Upper panel, whole MSN. Lower panel, model zoomed with one of the sections selected (a spine head).
(TIF)

Figure S12 **Comparison between electrical and biochemical calcium.** A, timecourse of calcium in a spine head of the electrical model. B, timecourse of calcium in the biochemical model of the corresponding spine head. The “biochemical” calcium is calculated using the equation 7 and approximates the “electrical” calcium.
(TIF)
