# Supplementary figures and images for: Integration of Biochemical and Electrical Signaling-Multiscale Model of the Medium Spiny Neuron of the Striatum
Source: PLoS One. 2013 Jul 3;8(7):e66811. doi: 10.1371/journal.pone.0066811 (PMC3700997; doi:10.1371/journal.pone.0066811)

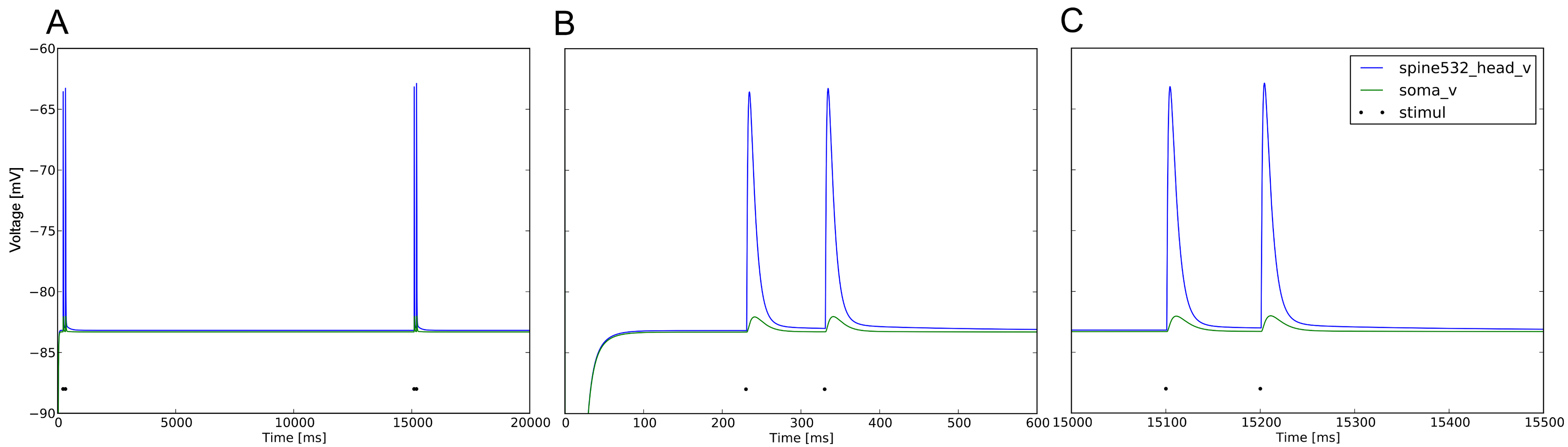

Supplement: Figure S1 — Two stimuli at different times on the same spine. The two pulses are applied at 100 ms and 15000 ms; A, complete timecourses; B, Zoom on the first stimulus; C, zoom on the second stimulus. (TIF) [file pone.0066811.s001.tif]

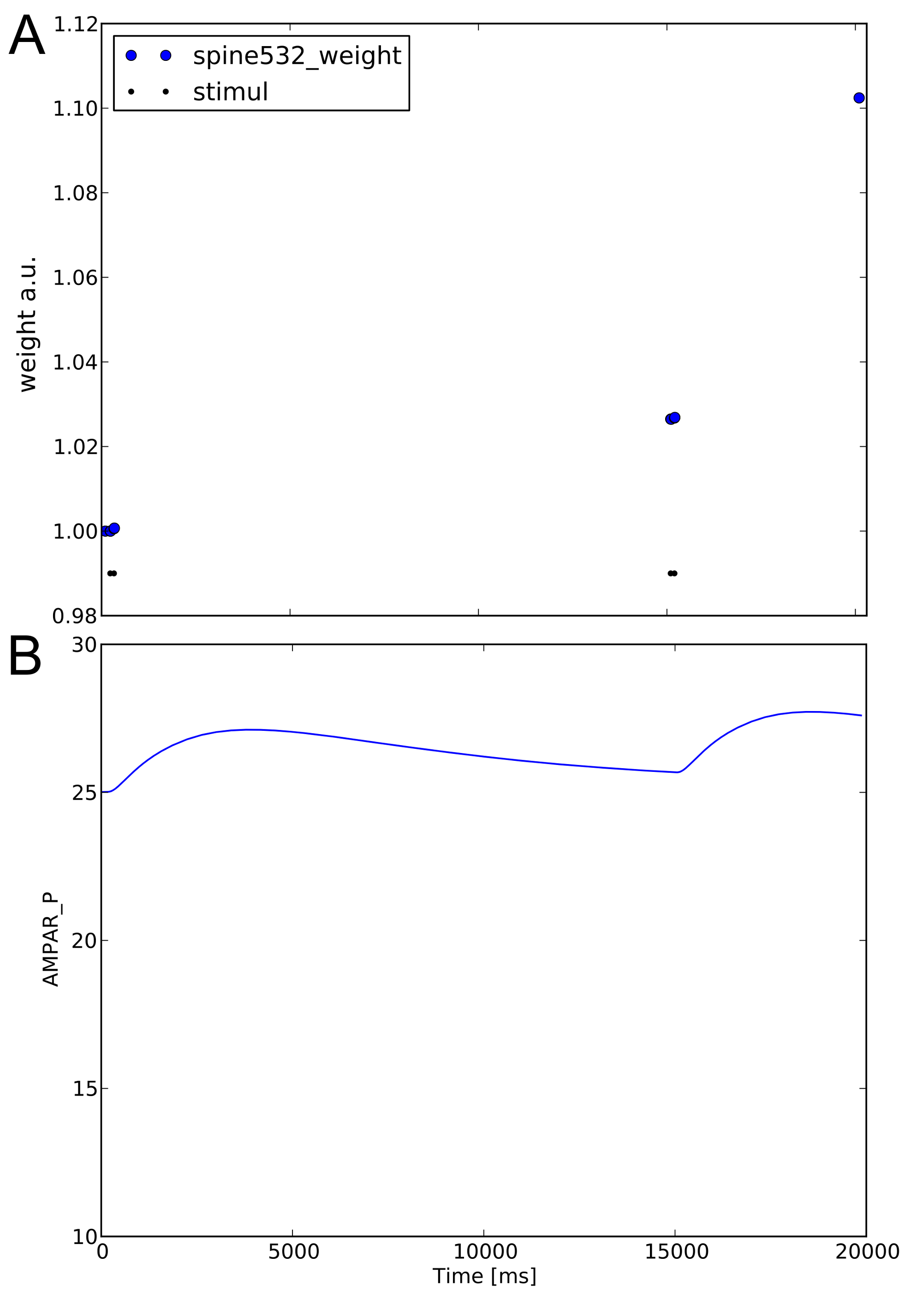

Supplement: Figure S2 — Effect of a short stimulation on the weight of the AMPA synapse. A, weight applied to the synapse on the electrical model. B, phosphorylated AMPA timecourse which is used to calculate the weight. A small stimulus triggers a minimal variation of the phosphorylated AMPA. (TIF) [file pone.0066811.s002.tif]

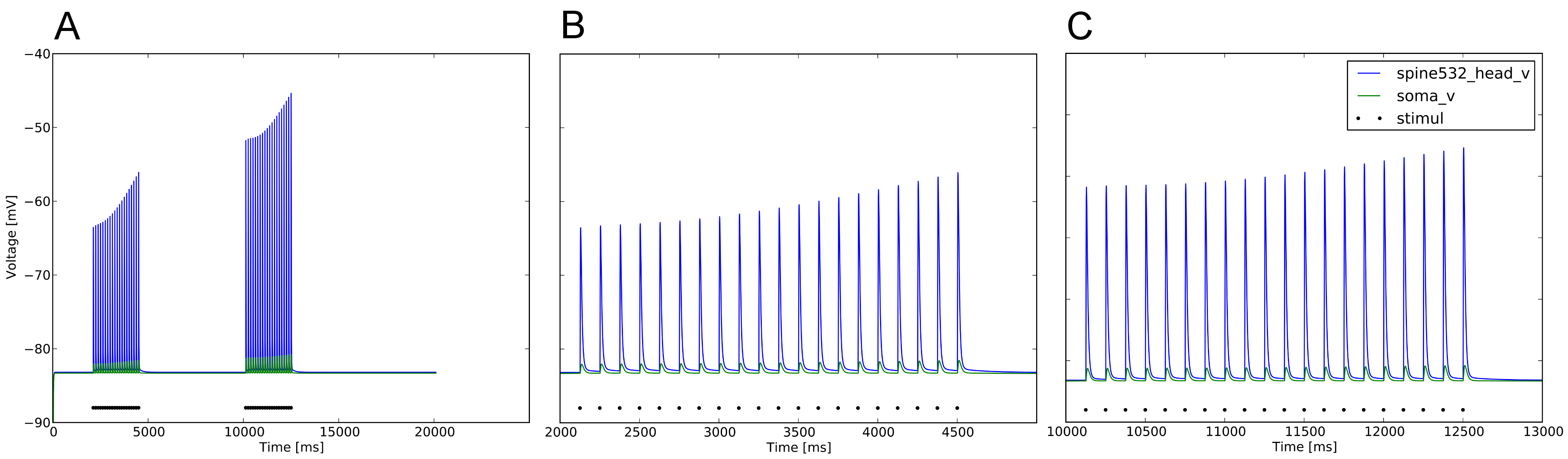

Supplement: Figure S3 — Single spine, response to a 8 Hz train stimulation. Response of the MSN model to a 8 Hz train stimulation. The weight increases during the stimulation. The response to the second train is larger in the spine because of the change of the synaptic weight connected with the biochemical model, which changes the number of AMPARs. A, complete timecourses; B, responses to the first train starting at 2100 ms; C, responses to the second train starting at 10100 ms. (TIF) [file pone.0066811.s003.tif]

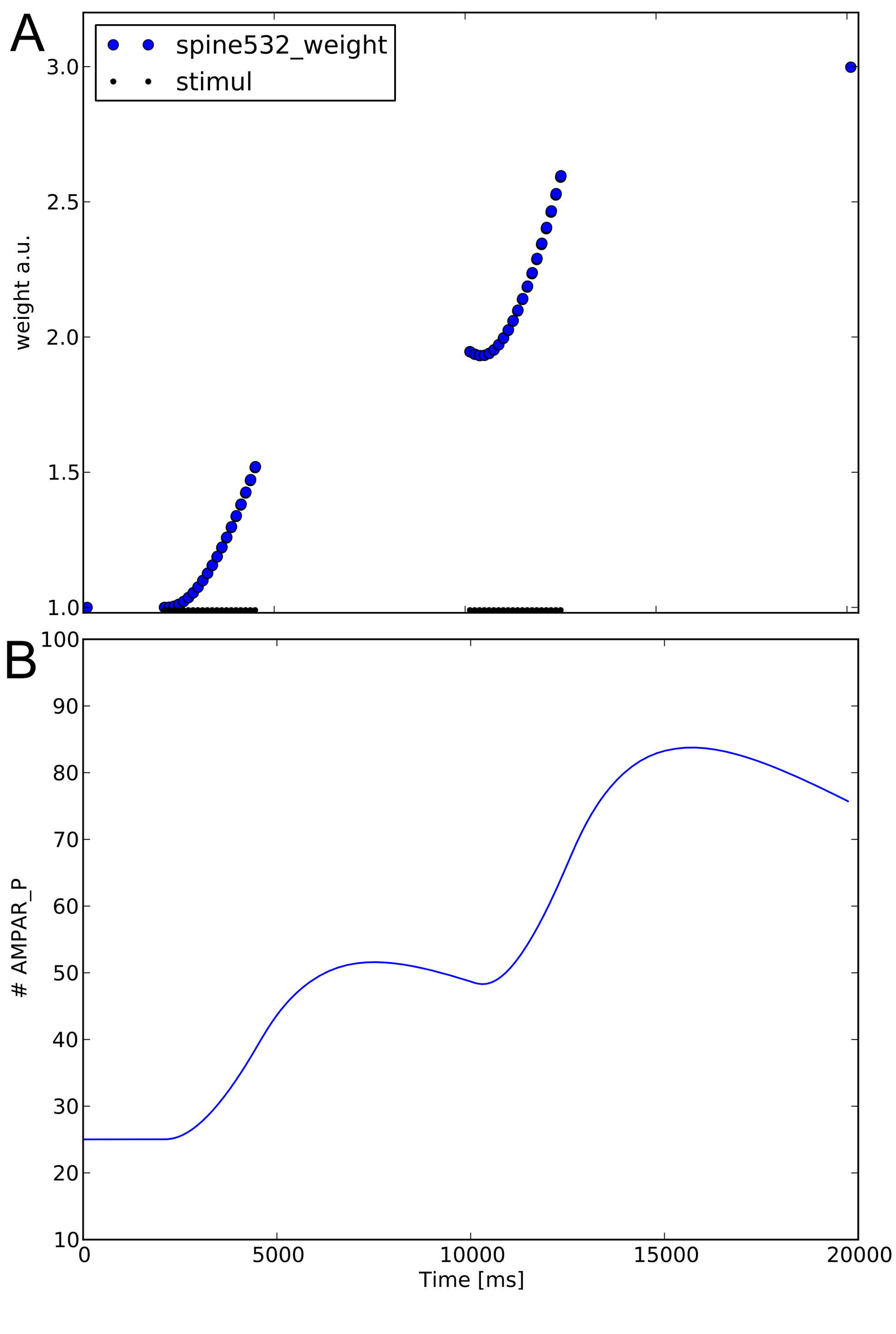

Supplement: Figure S4 — Effect of a large stimulation on the weight of the AMPA synapse. A, weight applied to the synapse on the electrical model. B, phosphorylated AMPA timecourse used to calculate the weight. A large stimulus triggers a significant variation of the phosphorylated AMPA, and the electrical response changes as a result. (TIF) [file pone.0066811.s004.tif]

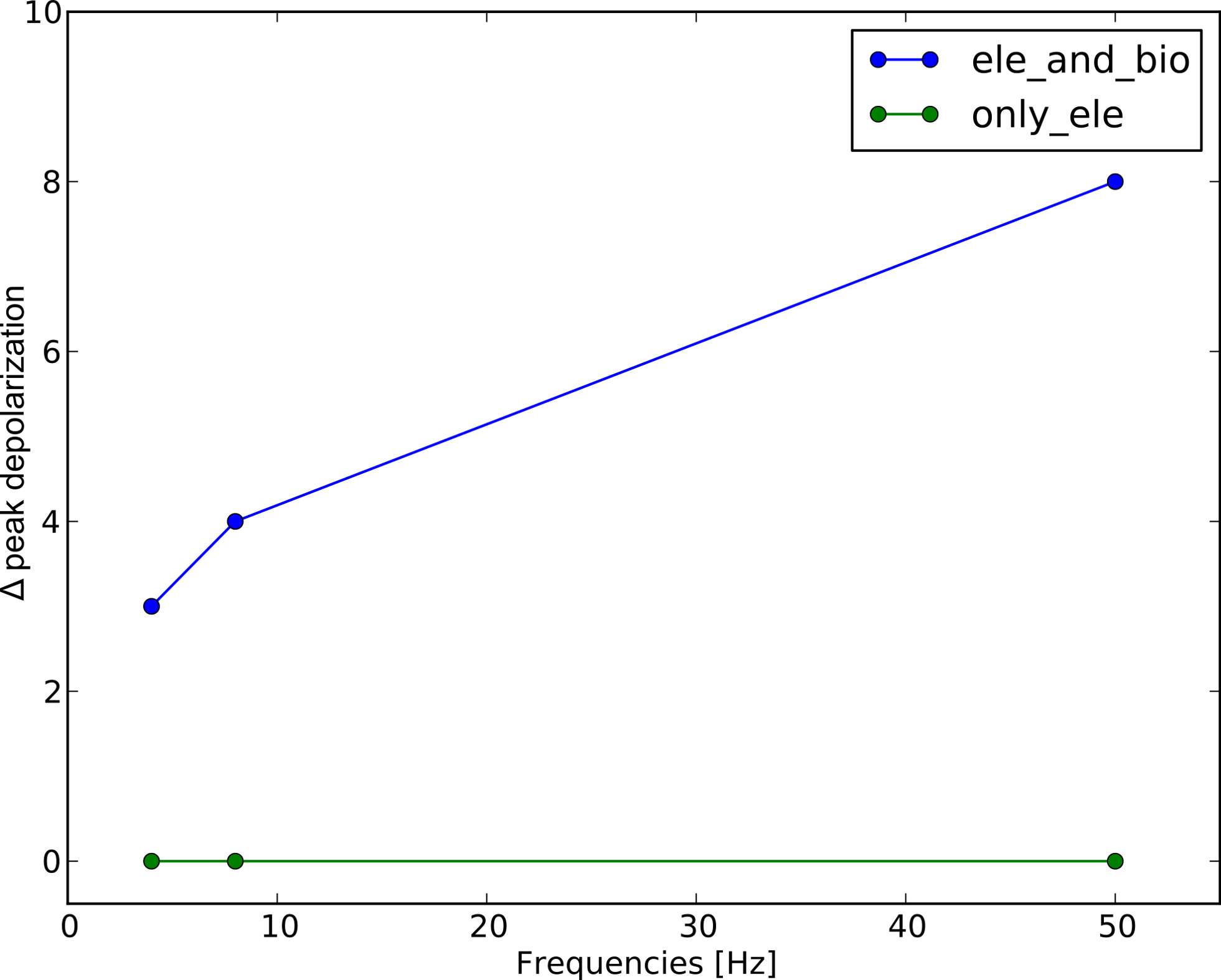

Supplement: Figure S5 — Difference of responses due to biochemical pathways. Double stimulations of a single spine (532) with increased frequencies. Y-axis represents the difference between the average peak voltage of responses to first and second train of stimulations. In the hybrid model, the second train is able to trigger an increased response compared to the first one (blue plot). This is due to the increased weight of the synapse mirroring increased number of AMPARs produced by the biochemical model. Not surprisingly, with the biochemical model turned off, there are no differences between the responses elicited by both trains (green plot). (TIF) [file pone.0066811.s005.tif]

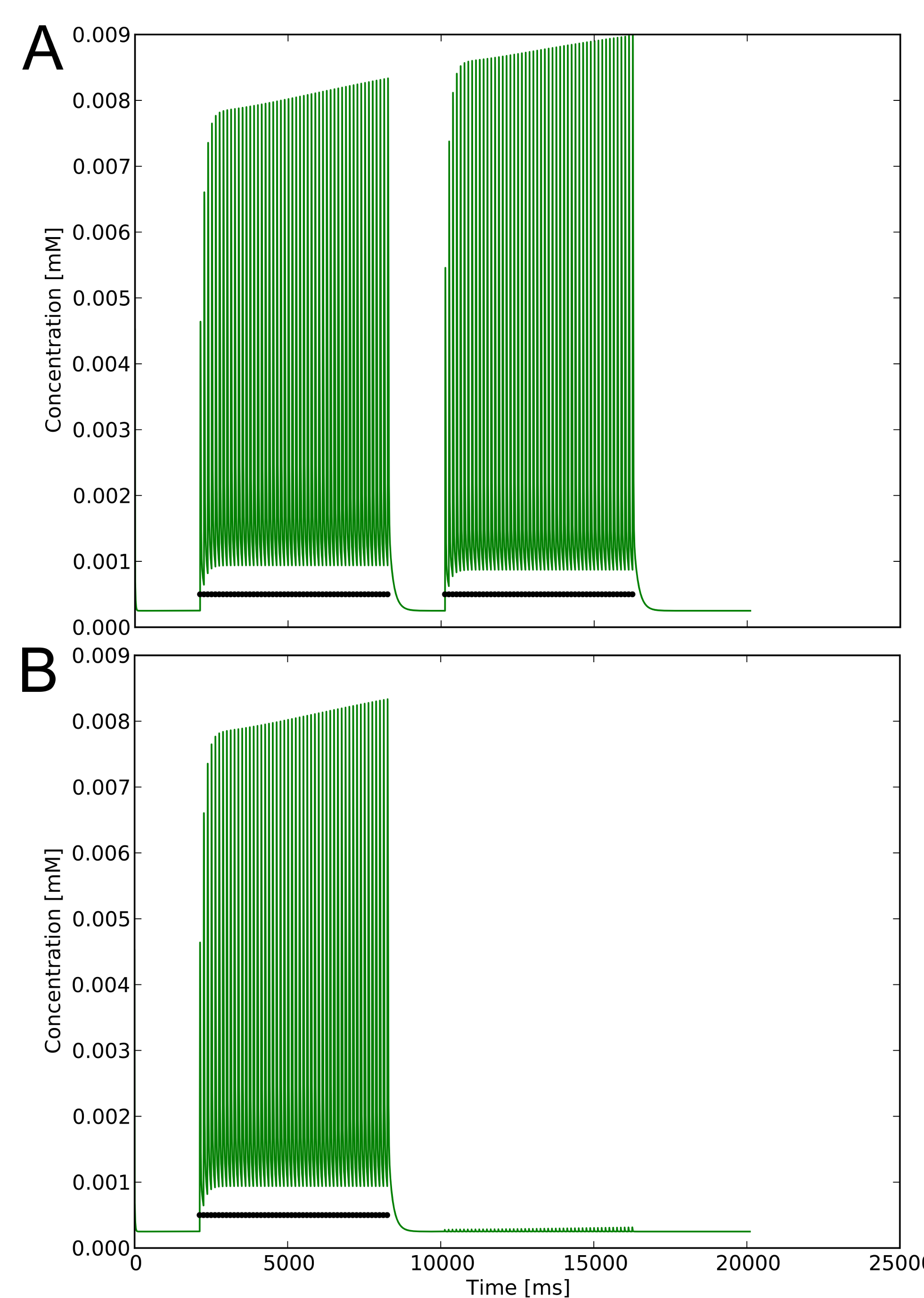

Supplement: Figure S6 — Calcium response in the adjacent spines. Calcium response of two adjacent spines. A, spine 559 receives two trains of stimuli. B, spine 560 only receives one train of stimuli. During the second train of stimuli, a small amount of calcium enters spine 560 due to the action of voltage-gated calcium channels activated by the stimulation of spine 559. (TIF) [file pone.0066811.s006.tif]

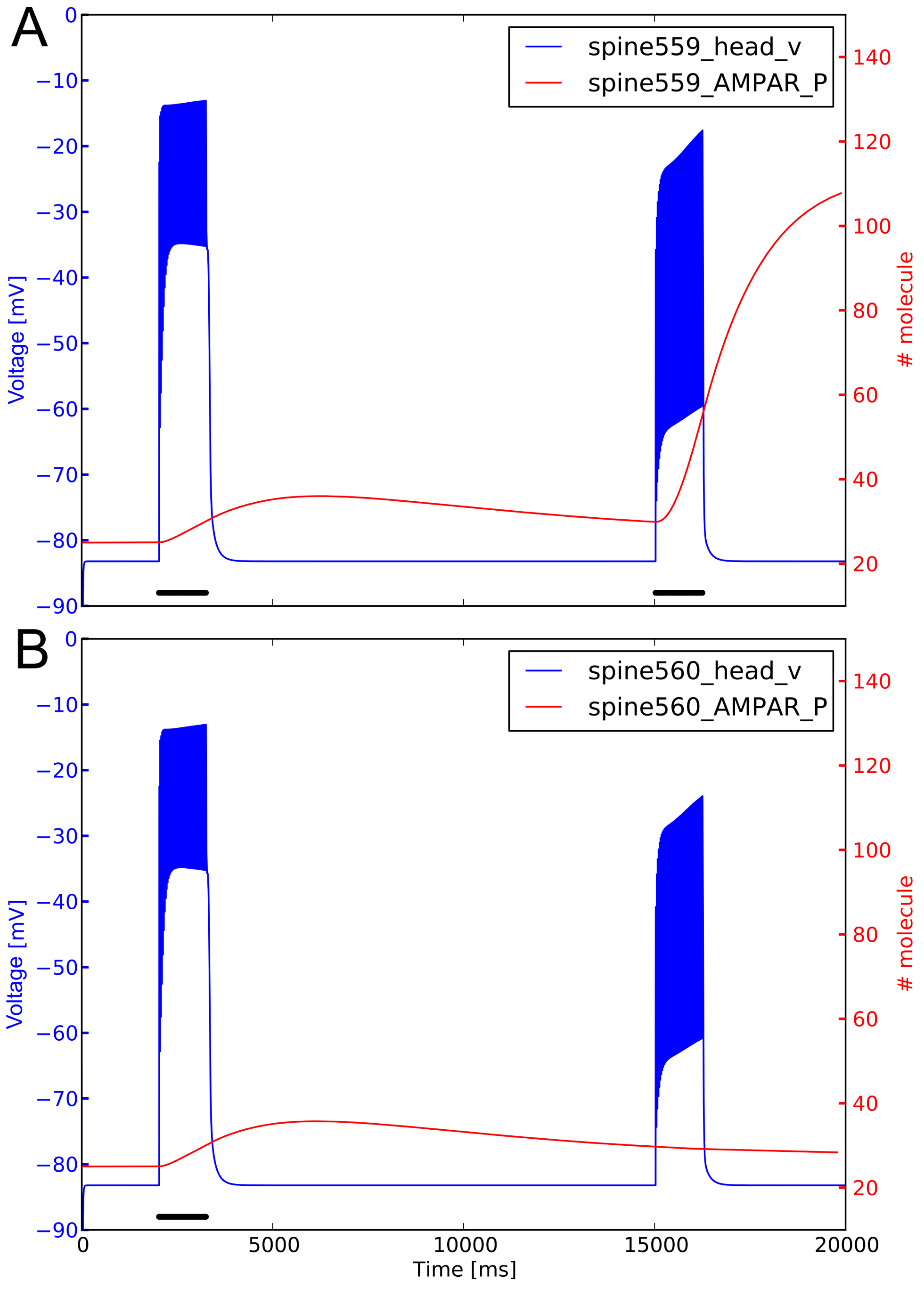

Supplement: Figure S7 — Difference of AMPARs phosphorylation in adjacent spines following a 40 Hz stimulation. A, spine 559 receives the first and the second trains of stimulation. B, spine 560 receives only the first train. (TIF) [file pone.0066811.s007.tif]

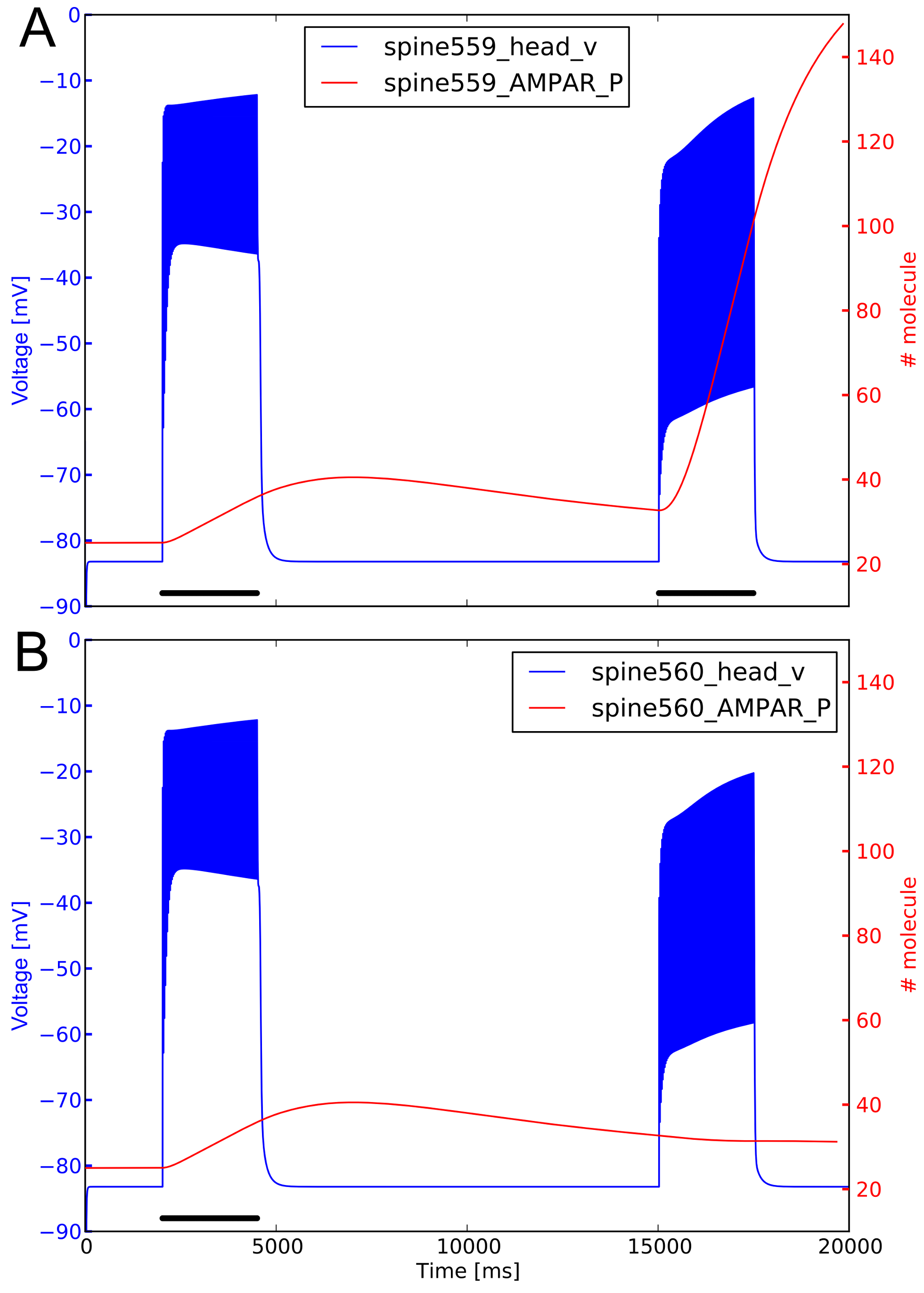

Supplement: Figure S8 — Difference of AMPARs phosphorylation in adjacent spines following a longer 40 Hz stimulation. A, spine 559 receives the first and the second trains of stimulation. B, spine 560 receives only the first train. (TIF) [file pone.0066811.s008.tif]

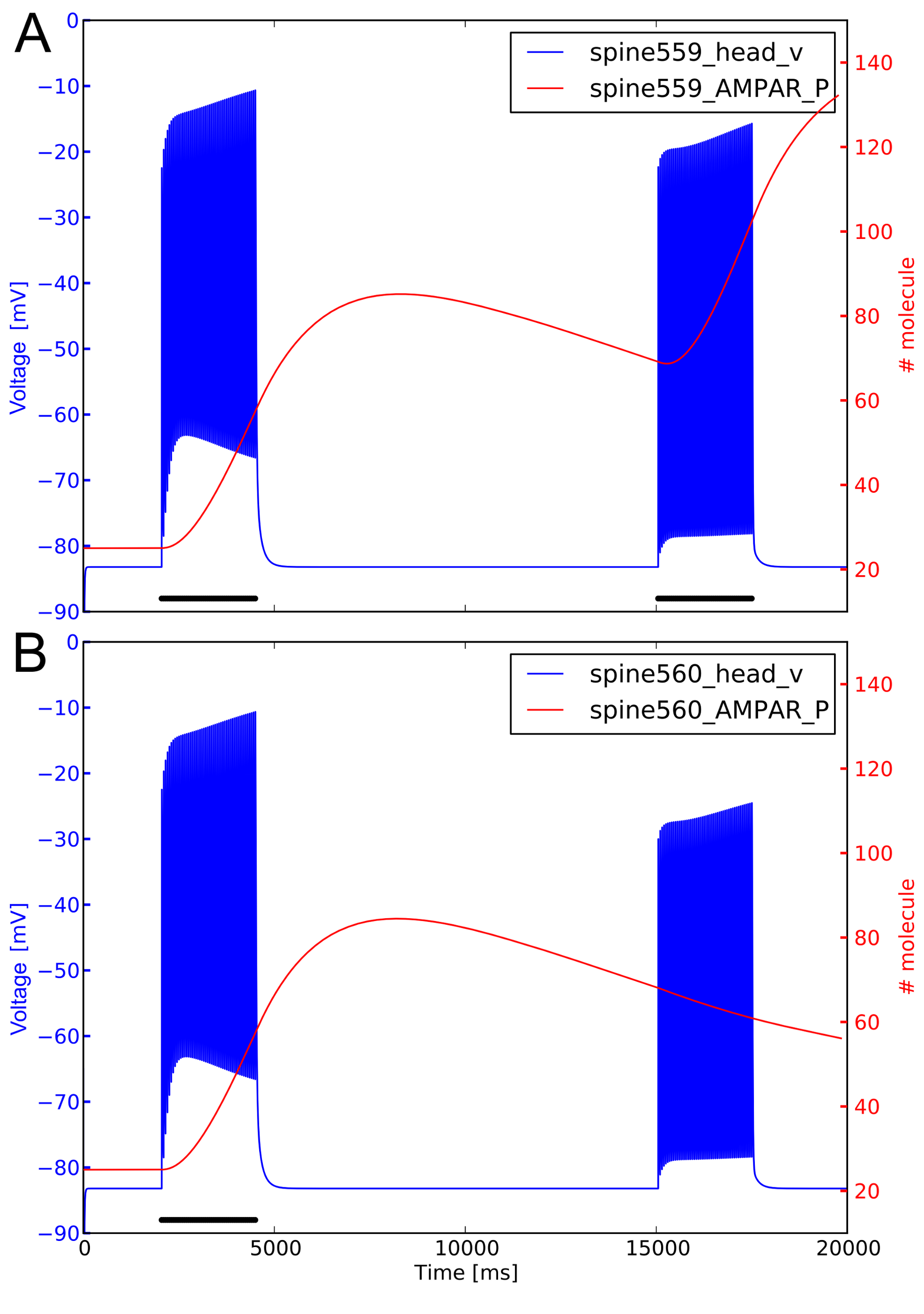

Supplement: Figure S9 — Difference of AMPARs phosphorylation in adjacent spines following a 20 Hz stimulation. A, spine 559 receives the first and the second trains of stimulation. B, spine 560 receives only the first train. (TIF) [file pone.0066811.s009.tif]

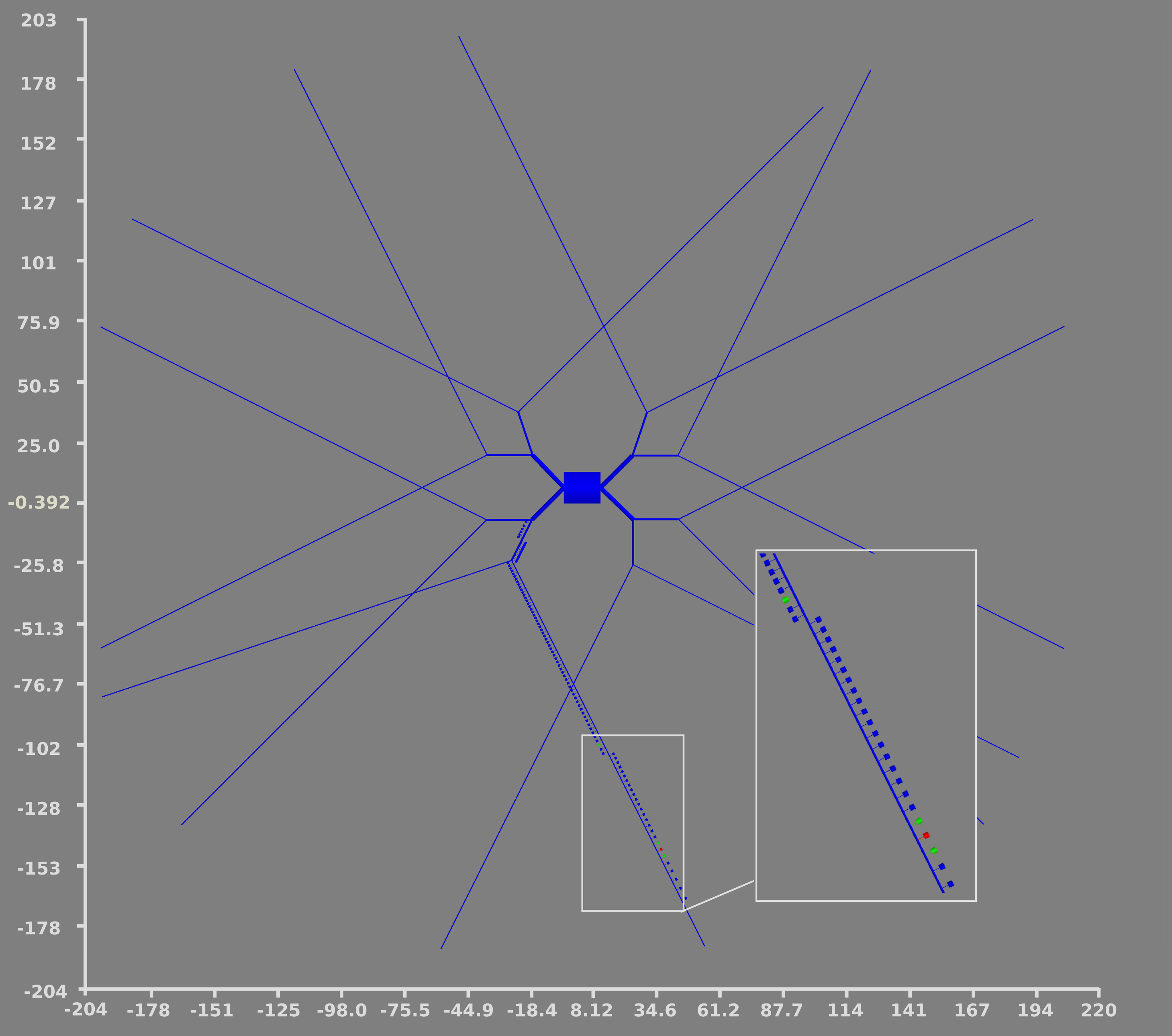

Supplement: Figure S10 — Tracking the effect of biochemical depolarization on adjacent and distant spines. Spines stimulated in one branch. Spine number 97, in red, is directly stimulated with two trains, while spines 75, 96 and 98, in green, are monitored to assess the influence of the electrical depolarization on the biochemical calcium. The axes are in . (TIF) [file pone.0066811.s010.tif]

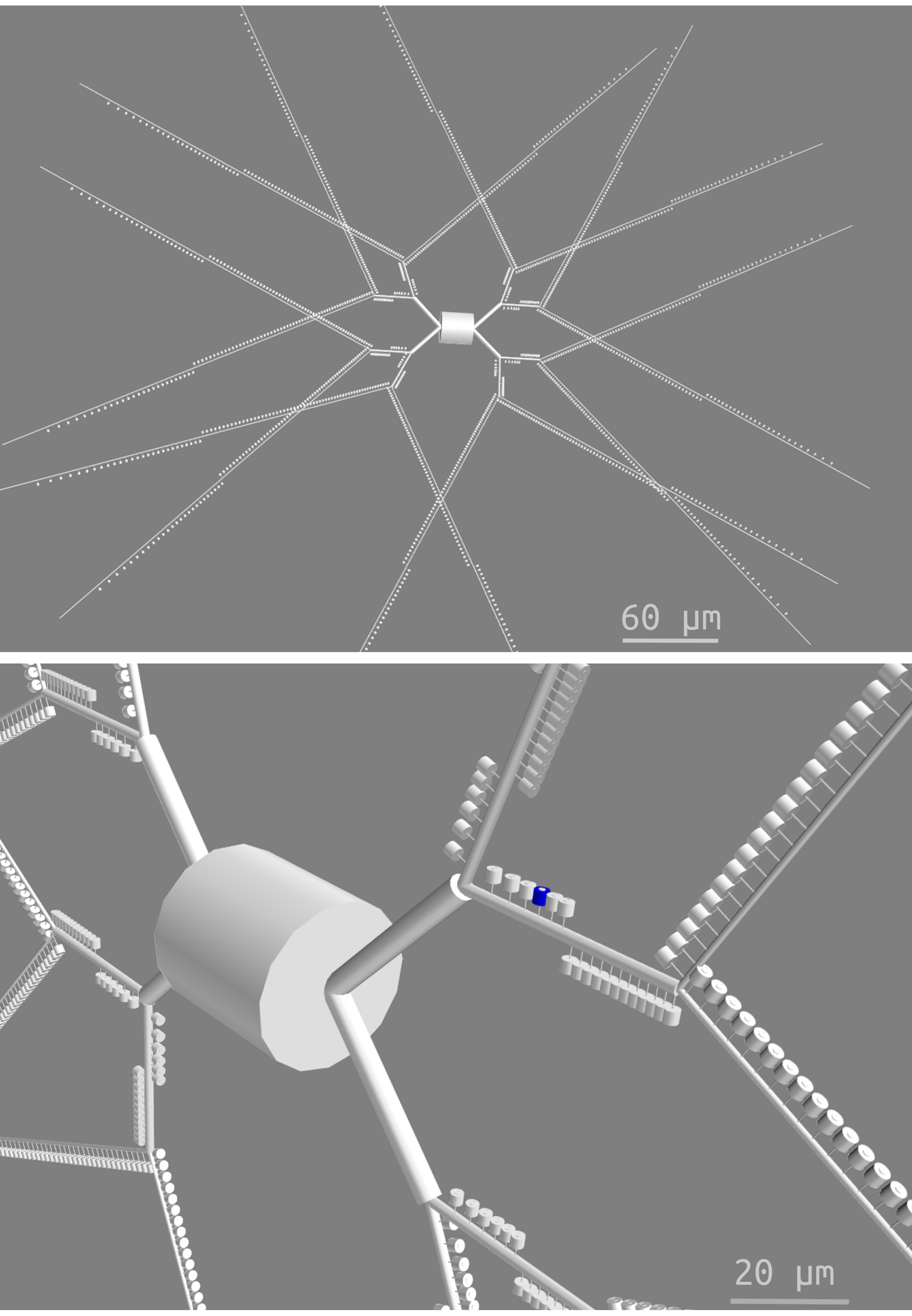

Supplement: Figure S11 — The multiscale MSN model. Multiscale MSN model rendered with Neuronvisio [78]. Upper panel, whole MSN. Lower panel, model zoomed with one of the sections selected (a spine head). (TIF) [file pone.0066811.s011.tif]

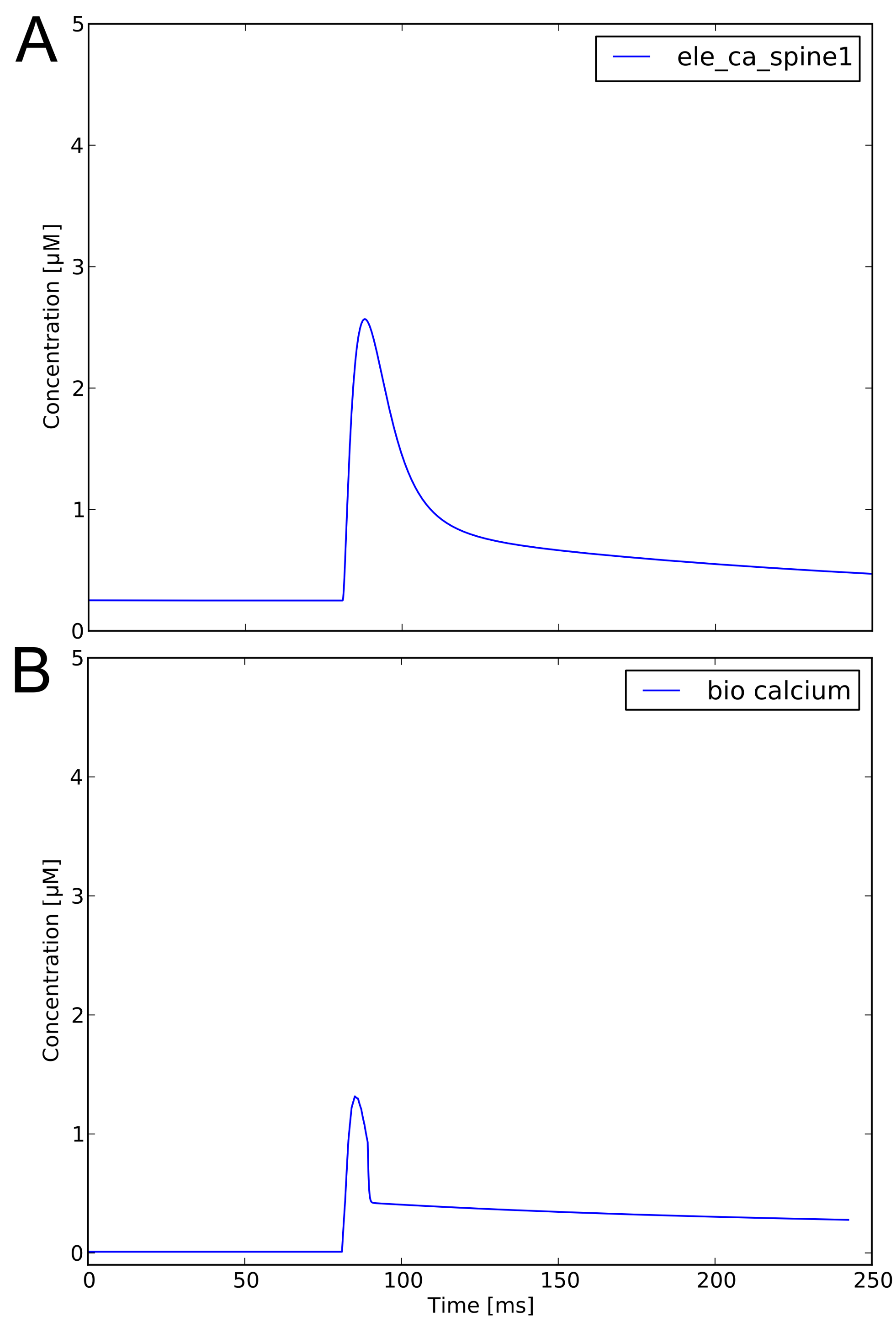

Supplement: Figure S12 — Comparison between electrical and biochemical calcium. A, timecourse of calcium in a spine head of the electrical model. B, timecourse of calcium in the biochemical model of the corresponding spine head. The “biochemical” calcium is calculated using the equation 7 and approximates the “electrical” calcium. (TIF) [file pone.0066811.s012.tif]
